# The GLP-1R agonist semaglutide reshapes pancreatic cancer associated fibroblasts reducing collagen proline hydroxylation and favoring T lymphocyte infiltration

**DOI:** 10.1186/s13046-024-03263-w

**Published:** 2025-01-20

**Authors:** Chiara Cencioni, Silvia Malatesta, Virginia Vigiano Benedetti, Valerio Licursi, Livia Perfetto, Federica Conte, Danilo Ranieri, Armando Bartolazzi, Martina Kunkl, Loretta Tuosto, Alberto Larghi, Geny Piro, Antonio Agostini, Giampaolo Tortora, Vincenzo Corbo, Carmine Carbone, Francesco Spallotta

**Affiliations:** 1 Institute of System Analysis and Informatics “Antonio Ruberti”, National Research Council (IASI-CNR), 00185 Rome, Italy; 2 Department of Biology and Biotechnologies “Charles Darwin”, Sapienza University, 00185 Rome, Italy; 3 Istituto Pasteur Italia-Fondazione Cenci Bolognetti, Sapienza University, 00185 Rome, Italy; 4 Department of Translational Medicine, Catholic University of the Sacred Heart, 00168 Rome, Italy; 5 Institute of Molecular Biology and Pathology, National Research Council (IBPM-CNR), 00185 Rome, Italy; 6 Dipartimento Di Scienze Della Vita, Della Salute E Delle Professioni Sanitarie. Università Degli Studi “Link Campus University”, 00165 Rome, Italy; 7 Pathology Research Laboratory, Sant’ Andrea University Hospital, 00189 Rome, Italy; 8 Neuroimmunology Unit, IRCCS Santa Lucia Foundation, 00179 Rome, Italy; 9 Digestive Endoscopy Unit, Fondazione Policlinico Universitario Agostino Gemelli IRCCS, 00168 Rome, Italy; 10 Center for Endoscopic Research Therapeutics and Training (CERTT), Catholic University, 00168 Rome, Italy; 11 Medical Oncology, Department of Medical and Surgical Sciences, Fondazione Policlinico Universitario Agostino Gemelli IRCCS, 00168 Rome, Italy; 12 Department of Engineering for Innovation Medicine (DIMI), University and Hospital Trust of Verona, 37100 Verona, Italy

**Keywords:** Pancreatic ductal adenocarcinoma, Cancer associated fibroblasts, Diabetes, Obesity, Collagen deposition

## Abstract

**Background:**

Metabolic syndrome represents a pancreatic ductal adenocarcinoma (PDAC) risk factor. Metabolic alterations favor PDAC onset, which occurs early upon dysmetabolism. Pancreatic neoplastic lesions evolve within a dense desmoplastic stroma, consisting in abundant extracellular matrix settled by cancer associated fibroblasts (CAFs). Hereby, dysmetabolism and PDAC association was analyzed focusing on CAF functions.

**Methods:**

PDAC development upon dysmetabolic conditions was investigated in: 1) high fat diet fed wild type immunocompetent syngeneic mice by orthotopic transplantation of pancreatic intraepithelial neoplasia (PanIN) organoids; and 2) primary pancreatic CAFs isolated from chemotherapy naïve PDAC patients with/without an history of metabolic syndrome.

**Results:**

The dysmetabolic-associated higher PDAC aggressiveness was paralleled by collagen fibril enrichment due to prolyl 4-hydroxylase subunit alpha 1 (P4HA1) increased function. Upon dysmetabolism, P4HA1 boosts collagen proline hydroxylation, intensifies collagen contraction strength, precluding PDAC infiltration. Noteworthy, semaglutide, an incretin agonist, prevents the higher dysmetabolism-dependent PDAC stromal deposition and allows T lymphocyte infiltration, reducing tumor development.

**Conclusions:**

These results shed light on novel therapeutic options for PDAC patients with metabolic syndrome aimed at PDAC stroma reshape.

**Supplementary Information:**

The online version contains supplementary material available at 10.1186/s13046-024-03263-w.

## Background

Metabolic syndrome is a complex metabolic disorder characterized by the manifestation of at least three concomitant clinical conditions among hypertension, obesity, hyperglycemia, hypertriglyceridemia, and hypercholesterolemia [1–3]. It is a pathologic condition deserving particular attention due to its severity and diffusion especially in Western countries for its alarming impact on health and care costs [1–3]. According meta-analyses, metabolic syndrome increases susceptibility to different cancers and in particular to gastrointestinal (GI) cancers, among the top 10 tumors for morbidity and mortality globally [4]. Gastric and pancreatic cancers are the GI cancers with the lowest survival rate, about 5–20%, absence of routinely diagnostic screening protocols and limited availability of therapeutic options [5].

Among the clinical conditions determining metabolic syndrome, hyperglycemia and obesity represent two known risk factors for pancreatic cancer in association with smoking and chronic pancreatitis [6]. Interestingly, diabetes is not only associated with a higher risk to develop pancreatic cancer, but also might represent one of the first signs of its onset [7, 8]. This renders the association between diabetes and pancreatic cancer bidirectional and difficult to discern. Indeed, retrospective analyses suggested that impaired glucose tolerance and hyperglycemia appear 30–36 months prior pancreatic cancer diagnosis and almost 50% of newly diagnosed pancreatic cancer patients met the criteria for diabetes based on fasting glucose levels [7].

Pancreatic ductal adenocarcinoma (PDAC) is the type of pancreatic malignancy with the highest prevalence, predicted to become the second deadly tumor by 2030 [9–11]. It is a heterogeneous disease with a 5-year survival rate around 13%, due to late diagnosis, associated with the most recurrent genetic alteration in KRAS (90% of PDAC cases) [12]. From a histological point of view, atypical tumor glands, constituted by neoplastic epithelial cells, occur in dense desmoplastic stroma, a proper physical barrier promoting tumor growth and progression and contributing to hamper anti-tumoral response and therapeutics delivery [11–14]. The PDAC desmoplastic stroma is abundant in extracellular matrix (ECM) deposition, cancer-associated fibroblasts (CAFs), and disorganized endothelium, characterized by insufficient anti-tumoral response [11, 13, 14]. According transcriptomics analyses, different PDAC subtypes have been proposed to stratify patients [15, 16]. Of note, specific molecular subtypes according stroma organization have been described and classified as “normal” and “activated”, with the latter showing a worst prognosis [15].

Pancreatic CAFs (pCAFs) are responsible of ECM deposition, immune evasion/exclusion and support tumor growth contributing to generate the high desmoplastic stroma typical of PDAC [17, 18]. Indeed, pCAFs continuously remodel the tumor microenvironment (TME). They promote and harness the deposition of collagens, hyaluronan, and proteoglycans; secrete different paracrine factors, including growth factors, cytokines and chemokines, supporting tumor progression; and repress anti-tumoral response both recruiting immune suppressor cells and inhibiting cytotoxicity activity, by co-expressing antigen presenting molecules and immune checkpoint ligands [19, 20]. Furthermore, pCAFs might sense metabolic alterations associated with metabolic syndrome, since it has been proven that the stromal compartment might retain the so called “metabolic memory” established upon prolonged exposure to hyperglycemia [21, 22].

The enzymatic activity of prolyl 4-hydroxylase subunit alpha 1 (P4HA1), a procollagen-proline, 2-oxoglutarate 4-dioxygenase, is crucial for collagen polypeptide chain folding into stable triple-helical molecules and consequently for ECM deposition [23]. Its involvement in cancer biology has been already described so much that it can be accounted as a prognostic biomarker of different tumors [24, 25]. P4HA1 enzymatic activity was described in relation to cell proliferation, invasion, migration and metastasis development processes in different tumors, including breast cancer, prostate cancer, colorectal cancer, hepatocellular carcinoma, oral squamous cell carcinoma, melanoma, and glioma [26–32]. Instead, its function in PDAC stroma is still elusive and not properly investigated. Moreover, the effect of dietary and blood glucose control on the stroma have been barely characterized, although blunt reduction in caloric intake, without malnutrition, showed promising anti-tumorigenic effects [33]. Nevertheless, how dysmetabolism affects PDAC stroma organization deserves dedicated research to assess whether dysmetabolic-dependent “metabolic memory” acquisition affects PDAC stroma evolution, contributing to worsen prognosis and hinder therapeutic response. In this light, the dissection of the complex association between dysmetabolism and PDAC, with a specific focus on stroma, and in particular on P4HA1 role, could help to unveil novel targets for prevention, early diagnosis and intervention aimed at reducing incidence and improving PDAC prognosis. 

Interestingly, retrospective analysis on obese and diabetic patients revealed a chemo-preventive potential of bariatric surgery or treatment with anti-diabetic and/or anti-hypertension medicaments with risk reduction of PDAC development [34–36]. Instead, no clear results are available at moment about the chemo-adjuvant potential of the same therapeutic strategies in PDAC. For instance, it has been shown that anti-diabetic drugs counteracting insulin resistance (i.e. metformin) seem to decrease PDAC risk, whereas the ones aimed to circulating insulin enhancement (i.e. insulin analogs) manifest an increased risk [36–39]. Data on a novel class of anti-diabetic medicaments (i.e. incretin-based analogues) are still controversial and barely characterized [40–42]. In this light, there is an unmet need to specifically conduct dedicated studies on this aspect. In perspective, the repositioning of this class of treatment for chemo-adjuvant protocols is of particular interest, since all these medicaments are well-characterized from a pharmacological and toxicological point of view as well as adjustment strategies in case of comorbidities, ultimately reducing failure risk and costs.

Here, we investigated the effect of dysmetabolism on PDAC development in a pre-clinical PDAC model with a specific focus on the stromal compartment. Our results have been analyzed taking advantage of a bio-bank of human pCAFs (hpCAFs) isolated from chemotherapy naïve PDAC patients with or without an history of metabolic syndrome to further evaluate its role in shaping TME. Moreover, we specifically analyze how semaglutide treatment, an incretin analogs, with weight-loss and anti-diabetic properties [43, 44], might shape the stroma towards a more permeable and immune permissive microenvironment. Taken together, our results suggest a novel therapeutic potential to be exploited in chemo-adjuvant strategy for the treatment of PDAC patients affected by metabolic syndrome.

## Methods

### Sex as a biological variable

Our study examined male mice because male animals display higher and faster response to metabolic alterations induced by high fat diet (i.e. higher body weight gain, blood glycemia increase) than female animals [45]. Moreover, male mice allowed to the research group to adhere to 3 r’s principles for animal studies, because the hyperglycemia threshold of 200 mg/dl fixed for the experimental plan and to proceed with semaglutide treatment was reached faster and more consistently (10–11 weeks) with a less number of animals compared to female mice, which usually are protected against HFD-induced metabolic alterations [45], dilating time to achieve hyperglycemia condition. Our study examined male and female human samples, as reported in Supplementary Table 1, and similar findings are reported for both sexes.

### PDAC mouse model

Male C57BL/6 WT mice (n = 65) were obtained from Catholic University of Sacred Heart animal facility. Soon after weaning, at 35 days, mice were randomly divided in 2 groups and fed with low fat rodent chow (LFD- 10 kJ% fat, 20 kJ% protein, 70 kJ% carbohydrates – D12450J Ssniff—Germany; n = 26) and high fat diet (HFD- 60 kJ% fat, 20 kJ% protein, 20 kJ% carbohydrates – D12492 Ssniff—Germany; n = 39) for 10 weeks before organoid orthotopic implantation and for the entire duration of the experiment. At week 10, when mice reached the threshold fasting blood glycemic value of 200 mg/dl, the insulin-resistance onset sign, an additional group was derived from the HFD one to receive subcutaneously once a week for 6 weeks Semaglutide (SEMA- 30 nmol/kg; Adipogen), an anti-dysmetabolic agent acting as GLP1-RA (n = 18). All mice were housed and treated in compliance with the European Council directives (No.86/609/EEC) and with NIH Guide for the care and use of laboratory animals (eight edition). The experimental plan was approved by the Animal Ethic Committee of the Catholic University of Sacred Heart of Rome (permit number: D.M. 593/2019-PR). Harvested organoids were mechanically dissociated, resuspended, orthotopically transplanted as previously described [46]. High resolution ultrasound imaging scan was performed to monitor dimension of developing tumor lesions using Vevo 3100 System (VisualSonics, Amsterdam, The Netherlands).

### Mouse monitoring and blood analyses

Body weight and food intake were recorded weekly. Fasting glycaemia was measured at experiment starting point and every month using a standard glucometer (GlucoGmeter, Menarini Diagnostics) on blood collected from mouse tail vein. The oral glucose tolerance test (OGTT), was performed after a fasting period of 6 h by administering glucose (2 g/kg) by oral gavage. Blood samples were obtained before glucose administration and at 15, 30, 60 and 120 min afterwards by blood collection from mouse tail vein, measuring glucose concentration by glucometer.

### Western blotting

Western blotting was performed by standard procedures. Laemmli buffer was used to obtain cell lysates. RIPA buffer was used to obtain mouse pancreatic tissue lysates. Nitrocellulose blotting membranes were probed with the following antibodies: pAkt (Cell signaling); total Akt (Cell signaling); P4HA1 (Abcam); GLP1-R (Bioss); Myc-Tag (Cell signaling); vinculin (Sigma) and α-Tubulin (Cell signaling). The list of all used antibodies is provided in Supplementary Table 2. Protein detection was obtained by ECL (Amersham, GE Healthcare Boston, MA, USA) and development was performed by UVITEC reader (Eppendorf Srl, Hamburg, Germany).

### Histology

Pancreatic tissue was embedded in optimal cutting temperature (OCT). 12 μm cryosections were analyzed for hematoxilin/eosin (H&E), immunofluorescence, immunohistochemistry and picrosirius red staining (BioOptica) according standard procedures. A pathologist, blinded to mouse group information, determined pancreatic cancer grade observing H&E stained slides. PanIN lesion total number of different histologic grades (from low-grade PanIN to grade 2 (G2) carcinoma) was annotated. When different grade PanIN lesions were observed in the same pancreatic duct, they were counted separately. Abrupt transition of normal ductal epithelium to highly atypical epithelial cells was considered as a sign of carcinogenesis. Immunofluorescence was performed using the following primary antibodies: CD3 (Abcam), CD8 (Abcam), E-cadherin (Invitrogen), Pan-citokeratin (Invitrogen), alpha-SMA (Invitrogen), P4HA1 (Abcam), and Hydroxyproline (Cell signaling). The following primary antibodies were employed for immunohistochemistry analyses: Col1A1 (Santa Cruz) and Hydroxyproline (Cell signaling). The list of all used antibodies is provided in Supplementary Table 2. H&E, picrosirius red staining and immunohistochemistry were analyzed using the EVOS XL Core microscope (Invitrogen). Signal quantification was calculated using Fiji ImageJ software. Immunofluorescence was analyzed by Nikon Eclipse Ti2 confocal microscope and Z stack images were processed by NIS Element AR 5.30 software (Nikon Europe BV). The mean fluorescence intensity (MFI) was quantified by Fiji ImageJ software on pictures acquired with same settings. At least 15 fields were considered.

### Total RNA extraction, sequencing and bioinformatics analysis

For next generation sequencing (NGS) RNA was isolated from mouse pancreatic neoplastic lesions at T1 (30 days; LFD mice n = 3; HFD mice n = 4) and T2 from orthotopic PanIN lesion transplantation (90 days; LFD mice, HFD mice and SEMA mice; n = 4 for each experimental group) by miRNeasy micro Kit (Qiagen) combined with on-column DNase digestion (DNase-Free DNase Set, Qiagen) to avoid contamination by genomic DNA according manufacturer’s instruction. Then, RNA integrity was verified by BioAnalyzer 2100 (Agilent) and only RNA samples with a RNA integrity number (RIN) > 7 were processed for cDNA library construction. Sequencing was performed on rRNA-depleted total RNA (50 M PE reads, 7.5Gbp) on an Illumina HiSeq 2000 platform. The resulting raw reads were assessed for quality by FastQC and preprocessed with FASTQ Toolkit v.2.2.5 for adapter trimming and low quality (Q < 30) filtering of the reads.

Then reads were aligned to the reference Genome assembly version GRCm39 using salmon aligner (v.1.10) [47] using a decoy-aware transcriptome index with k-mers of length 31 and normalizing for local GC content. GENCODE Gene Set version M27 was used for gene annotation. Transcripts were merged to genes using Bioconductor [48] R (v.4.3.1) package tximport (v1.28.0) [49]. To identify differentially expressed genes (DEGs), data were filtered to remove from the analysis the genes having > 10 counts for at least 3 samples. Gene-level normalization and differential expression analysis were performed with R package DESeq2 v.1.40.2 [50]. Principal component analysis was performed using R base prcomp function on regularized logarithm transformed gene expression levels. R packages ggplot2 v.3.5.0. and ComplexHeatmap v.2.16.0 were used to generate heatmaps. Gene ontology and KEGG Pathways analysis on genes regulated by HFD at T1 and T2, as well as by SEMA (± 1 log2 fold change, fdr < 0.05) was performed using DAVID (https://david.ncifcrf.gov/). Broad Institute gene set enrichment analysis (GSEA) [51] was used to assess the enrichment of the ranked gene expression profiles versus the curated “Hallmark” and selected Gene Ontology C5 gene set collections from the Broad Molecular Signatures Database (MSigDB) v.7.5.1. Normalized enrichment score (NES) was also calculated with GSEA, by considering differences in pathway size (i.e., gene set size) and allowing for comparisons between signatures within the analysis. A network-based deconvolution approach was performed with Imsig R package v.1.1.3 [52] that contains functions for profiling the tumor microenvironment from RNAseq data and specific immune gene signatures derived by transcriptomics data from solid tumors. The Imsig pipeline was applied to RNAseq normalized expression matrix and T lymphocyte signature from Imsig package was used to estimate T cell infiltration levels, while for estimating the stroma component the pancreas mesenchymal stromal cell gene set from MSigDB was used (cell type signature collection C8, systematic name M39175, based on single-cell RNAseq data from pancreas) [53]. A correlation cut-off of 0.7 was used, to enrich the prediction of relative abundance of immune or stromal cells by filtering off poorly correlated genes of the signatures. Imsig resulting scores were subsequently analyzed by multiple comparisons of groups of samples performing a Tukey's HSD after one-way-ANOVA.

Moreover PDAC data (transcriptomics and proteomics) from 140 patients, available at the Clinical Proteomic Tumor Analysis Consortium (CPTAC) repository (February 2023), were downloaded for CPTAC analysis using the Jupiter Notebook interface version 6.4.12. Data were subsequently normalized by sample-specific z-scoring (centered on the median). Next, the fgsea R package (1.24.0) was used to perform GSEA over the ranked proteomic data using the CAF infiltration and the stromal activation signatures [15, 16]. Patients were stratified according to the individual signature enrichment (YES: NES > 2 and adjusted p-value < 0.05). P4HA1 box plots were produced by comparing the transcript level of the gene in different stratification groups by t-test on transcriptomic data and visualized using the ggplot2 R.package (3.4.4).

### mRNA extraction and qRT-PCR

RNA was extracted from hpCAFs (approximately 10^6^ cells) or 2/3 cryosections of pancreatic lesion tissue using Trizol (Invitrogen) according to manufacturer's instruction. cDNA synthesis for quantitative real-time PCR (qRT-PCR) was carried out with LunaScript RT Super Mix (NEB) according to the manufacturer's protocol. All reactions were performed in 96-well format in the QuantStudio 3 Real-Time PCR System (Applied Biosystems) using Luna Universal qPCR Master Mix (NEB). For each gene of interest, qRT-PCR was performed as follows: each RNA sample was tested in duplicate and p0 (human and mouse) was used to normalize transcript abundance. mRNA expression levels were calculated by Comparative Ct Method by using the Applied Biosystem software (Applied Biosystem, CA, USA) and were presented as fold induction of transcripts for target genes. The following primers were used: Col1A1, Col4A1, Col6A1, Col3A1, Col5A1, P4HA1, and p0 and listed in Supplementary Table 3.

### Patients

The current study enrolled 30 PDAC patients (see Supplementary Table 3) naïve for chemotherapy undergoing pancreatic eco-endoscopy. Clinical information was collected for each patient and included age, sex, BMI, diabetes, and medical treatments. All data were collected and analyzed anonymously. All patients were enrolled after ethical committee approval and informed consent according to standard hospital procedures (permit n: CE ID 2179). Investigations were conducted according to the principles expressed in the Declaration of Helsinki.

### hpCAF isolation

Primary human pancreatic cancer associated fibroblasts (hpCAFs) were obtained from the small-size specimens derived from patient eco-endoscopy of non-dysmetabolic (ND) and dysmetabolic (D) donors. During the progress of this work and in consequence of their different growth rate, not all primary isolated cells could always be utilized. Consequently, not all the experiments were performed with the same isolated cells. After enzymatic digestion with Collagenase II (5 mg/ml; Gibco), Dispase (2 mg/ml; Gibco) and DNAse I (100 ng/ml; Merck), a cell population with fibroblast morphology was obtained and cultured in IMDM, 20% FBS, 1% Pen/Strep (Gibco), 1% Glutamine (Gibco), EGF (0.01 μg/ml; Cell Guidance) and bFGF (0.01 μg/ml; Cell Guidance), barcoded, frozen and, when necessary, randomly chosen among those available between the two groups. However, all 30 strains were assayed for proliferation. On average 4–5 independent randomly chosen cellular strains, from each group, were used in all the experiments. D-hpCAFs derived from newly diagnosed diabetic patients were excluded from analyses. To avoid senescence, cells were passaged no more than 4–8 times before assay. When required, hpCAFs were exposed to the following compound: Semaglutide 240 nM (Adipogen); 1,4-DPCA 40 μM (Cayman Chem).

### Flow cytometry analysis

Flow cytometry analysis was performed on a CytoFLEX S (Beckman Coulter, USA). Fluorescence staining was achieved with the following antibodies: CD29, CD44, CD31, EpCAM, CD45 listed in Supplementary Table 2. Gating strategy and identification of hpCAFs: within total acquired events, first the debris (low FCS) was excluded, followed by IgG control antibody signal. Flow cytometry data were analyzed using CytExpert software (Beckman Coulter).

### P4HA1 genetic manipulation

P4HA1 overexpression was achived by lentiviral infection. Briefly, ND-hpCAFs were infected for 16 h with lentiviral particles expressing P4HA1 (Lenti_P4HA1—Origene) or the empty vector (Lenti_EMPTY—Origene). Cells were allowed to recover in complete fresh medium for additional 48 h. Afterwards, infected ND-pCAFs were collected to evaluate P4HA1 level by Myc-tag expression and were used for collagen disk contraction test. P4HA1 loss of function experiments were performed by small interference RNA (siRNA) and by CRISPR/Cas9 technology. For siRNA, D-hpCAFs were transfected by siRNA targeting human P4HA1 (100 nM, Santa Cruz). Scrambled RNA sequence was used as control. Transfection was performed by siPORT NeoFX Tranfection Agent (Ambion) according to the instructions of the manufacturer. For CRISPR/Cas9, D-hpCAFs were electroporated with two different sgRNAs (Synthego) designed to specifically target human P4HA1 gene or non-targeting control (NTC) and SpCas9 RNA (Trilink) (Supplementary Table 4). Electroporation was performed by Nucleofector 2b Basic Kit for Fibroblast (Lonza) with Nucleofector II (Amaxa). HpCAFs engineered for P4HA1 expression (down-modulation or over-expression) were exploited to perfom contraction assay soon after P4HA1 expression was checked by WB analysis.

### Contraction assay

Determination of hpCAF contraction ability was achieved by collagen I polymerization at pH 8. Specifically, a mix of 9 mg/ml Collagen I rat tail (Corning) solution in 0.02 N acetic acid was prepared. Then, 10^6^ hpCAFs suspended in 10 × DMEM (Gibco), containing NaHCO3, Hepes and NaOH buffer solution were added by gentle mixing for each condition, pH was checked and adjusted with 1 M NaOH. The prepared mixture was distributed on transwells with 3 μm pore and incubated for 30 min in vibration-free incubator to allow polymerization. Soon after polymerization, complete medium was added and contraction was evaluated after 24–48 h, photographed by EVOS XL Core microscope (Invitrogen), and analyzed digitally measuring hydrogel diameter in pixels using Image J Fiji software. In specific experiments, D-hpCAFs were pre-treated for 24 h with Semaglutide 240 nM (Adipogen), or 1,4-DPCA 40 μM (Cayman Chem), or engineered for P4HA1 down-modulation by siRNA or CRISPR/Cas9 technology. Moreover, ND-hpCAFs were engineered for P4HA1 overexpression by lentiviral infection before performing contraction assay. For immune infiltration evaluation, 2 × 10^6^ human CD3^+^ purified lymphocytes, isolated from peripheral blood mononuclear cells (PBMCs) of buffy coat of anonymous healthy donors (Policlinico Umberto I, Sapienza University of Rome, Italy) by negative selection using EasySep Human T Cell Iso Kit (STEMCELL Technology) according to the manufacturer’s instructions, were resuspended in an appropriate volume, added above the hpCAF populated hydrogel and incubated overnight in the incubator. Healthy donors signed the informed consent and the Ethic Committee of Policlinico Umberto I (ethical code N., 1061bis/2019, 13/09/2019).

### ELISA assays

Fasting plasma level of GLP1 was determined by Mouse Glucagon Like Peptide 1 (GLP1) ELISA Kit (Bioss) according to the manufacturer’s instructions. Briefly, plasma was obtained from 5 LFD and 5 HFD mice, protein levels were quantified, and analyzed according assay sample preparation guidelines. Hydroxyproline determination was performed by Hydroxyproline Assay Kit (Sigma-Aldrich) according to the manufacturer’s instructions. 1.5 X 10^6^ ND- and D-hpCAFs were harvested after 24 h from last medium change. Moreover, at the end of contraction assay, hydrogels from different conditions were evaluated for hydroxyproline levels. Absorbance was measured by CLARIOstar plate reader (BMG Labtech).

### Study approval

All mice were housed and treated in compliance with the European Council directives (No.86/609/EEC) and with NIH Guide for the care and use of laboratory animals (eight edition). The experimental plan was approved by the Animal Ethic Committee of the Catholic University of Sacred Heart of Rome (permit number: D.M. 593/2019-PR). All patients were enrolled after ethical committee approval and informed consent according to standard Policlinico Gemelli Foundation-IRCCS of Rome hospital procedures (permit n: CE ID 2179). Human CD3^+^ purified lymphocytes were isolated from peripheral blood mononuclear cells (PBMCs) of buffy coat of anonymous healthy donors according Policlinico Umberto I, Sapienza University of Rome, Italy procedures.

### Data and code availability

The RNA sequencing datasets are publicly available at NCBI’s Gene Expression Omnibus (GEO) repository, under accession number GSE266899 located at https://www.ncbi.nlm.nih.gov/geo/query/acc.cgi?acc=GSE266899. Any additional information required to reanalyze the data reported in this work paper is available from the lead contact upon request. Further information and requests for resources should be directed to and will be fulfilled by the lead contact, Dr. Francesco Spallotta (francesco.spallotta@uniroma1.it).

### Statistical analysis

Statistical analyses were performed using GraphPad Prism software version 8. Sample sizes (n) were reported in the corresponding figure legend. For all analyses, the observer was blind to the identity of samples. Nonparametric student’s t-test was used to analyze variables. A value of p < 0.05 was deemed statistically significant. Mean values are indicated ± SEM. Kaplan–Meier curves related to P4HA1 expression according TCGA data on PDAC patients were generated using the Kaplan Meier plotter [54]. The scatter plot of the correlation analysis between T cell gene signature [55] and the P4HA1 gene expression profile in TCGA-PDAC patients was computed as follows. The specific gene signature was obtained by averaging the expression values of all genes included in that signature to visualize in a plot single patients as each black dot and linear regression as straight blue line estimating the relationship between the two variables above mentioned. The values “R” and “p” are the coefficient and the p values of the Pearson correlation test, respectively.

## Results

### HFD enhances collagen deposition and reduces T lymphocyte infiltration in neoplastic lesions contributing to a faster PDAC tumorigenesis

**Fig. 1:**
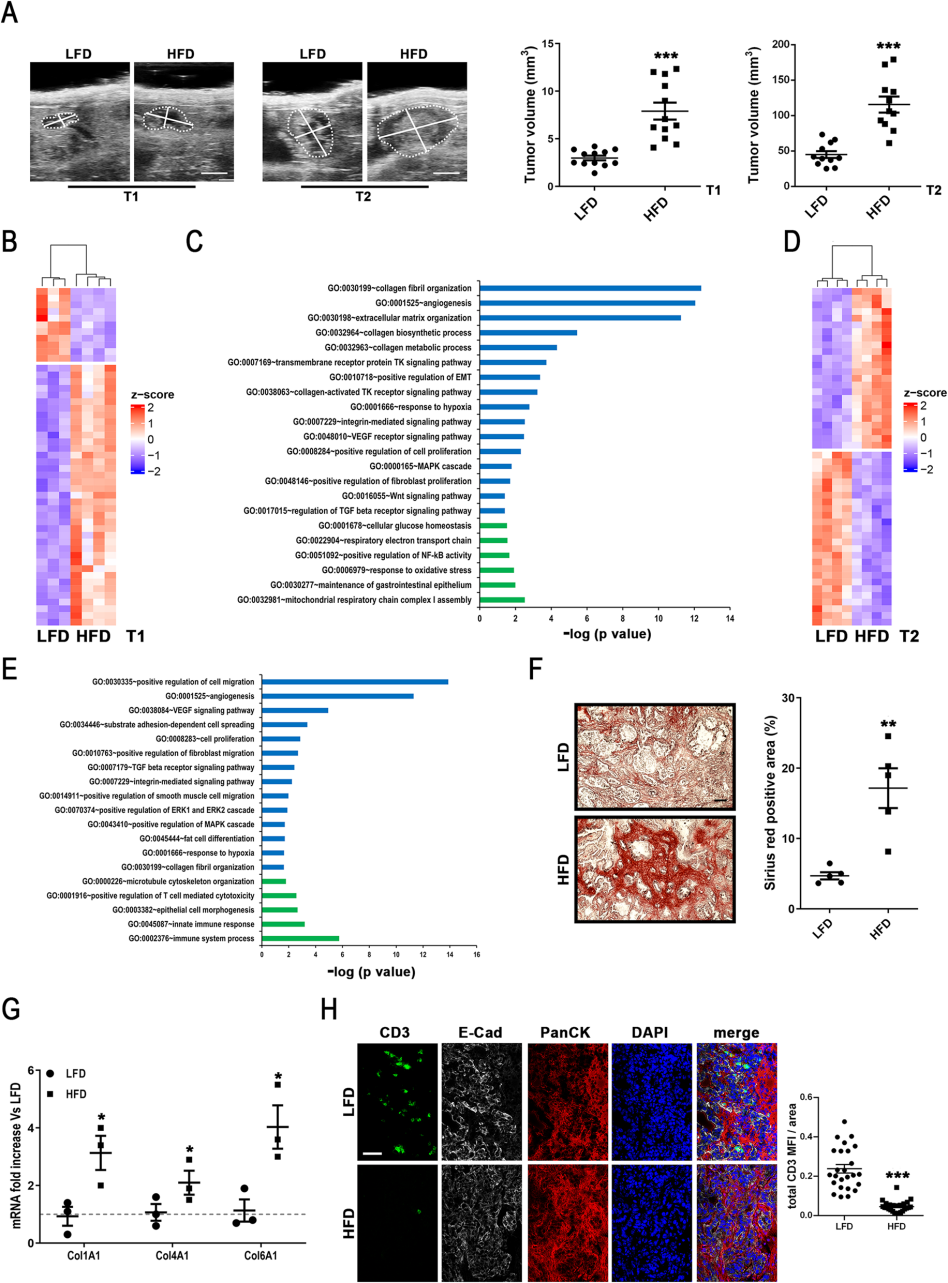
HFD mice showed a higher volume of pre-neoplastic pancreatic lesions paralleled by a higher collagen deposition, a reduced oxidative phosphorylation and decreased tumor infiltration. **A** Left panels: Representative endoscopic ultrasound images of LFD and HFD mice at T1 (30 days) and T2 (90 days) from KPC organoid injection. T1 original scale bar, 1.5 mm; T2 original scale bar, 1.8 mm; n = 10 for each group. Right panels: Tumor size measurement at time point T1 (left panel; n = 12 for each group; ***p < 0.001) and T2 (right panel; n = 11 for each group; ***p < 0.001) in LFD (black circles) and HFD (black squares) mice bearing pre-neoplastic lesions. **B** Heatmap showing the 50 most differentially regulated genes in LFD and HFD mice at time point T1 identified by total RNA sequencing analysis (n = 3 for LFD group and n = 4 for HFD group). Red and blue represent over- and under-expressed genes, respectively. **C** Gene ontology analysis of differentially regulated transcripts between LFD and HFD mice at time point T1. Up-regulated genes depicted in blue bars and down-regulated genes in green bars. **D** Heatmap showing the 50 most differentially regulated genes in LFD and HFD mice at time point T2 identified by total RNA sequencing analysis (n = 4 for each group). Red and blue represent over- and under-expressed genes, respectively.** E** Gene ontology analysis of differentially regulated transcripts between LFD and HFD mice at time point T2. Up-regulated genes depicted in blue bars and down-regulated genes in green bars. **F** Left panels: Representative picrosirius red staining images of pancreatic lesions at time point T2 in LFD (upper panels) and HFD (lower panel) mice bearing pre-neoplastic lesions. Right panel: Quantitative assessment of percentage of picrosirius red stained positive area in LFD (black circles) and HFD (black squares) mice at time point T2 (n = 5 for each group; **p < 0.01). **G** Col1A1, Col4A1 and Col6A1 mRNA analysis in LFD (black circles) or HFD (black squares) mice. Data expressed as fold increase of average Ct of LFD after subtraction of the housekeeping gene p0 signal (n = 3; *p < 0.05 HFD vs. LFD mice). **H** Left panels: Representative confocal microscopy images depicting LFD (upper panel) and HFD (lower panel) mouse cryo-sections at time point T2 probed by an anti-CD3 antibody (green, left panels), an anti-E-Cadherin (white, middle left panels), and an anti-pan cytokeratin (red, middle right panels). Nuclei were counterstained with DAPI (blue, right panels). On the right, merged images. Original scale bar, 50 μm; n = 5. Right panels: quantification of CD3 positive cell MFI on area in LFD (black circles) and HFD (black squares) mice (n = 25; ***p < 0.001). Data expressed as average ± SEM. Data analyzed by Kolmogorov–Smirnov test

PDAC onset and progression were investigated in wild type syngeneic mice receiving into pancreata pre-neoplastic lesion-derived organoids from PanIN-bearing Pdx1-Cre; Kras^+/LSL−G12D^; Trp53^+/LSL−R172H^ (KPC) mice, which well recapitulate human PDAC pathogenesis [56] and the stepwise progression of the spontaneous model including the loss of the wild-type allele of p53 as a pre-requisite for invasive tumors [57]. Specifically, soon after weaning, mice were randomly divided into two groups and fed either with low fat diet (LFD-18% energy intake from fat; n = 26) or high fat diet (HFD-60% energy intake from fat; n = 39) for 10 weeks. At 10 weeks of dietary regimen, metabolic parameter monitoring denoted a significant alteration in HFD mice compared to LFD ones. Indeed, as expected, HFD mice showed higher levels of body weight gaining (Suppl. Figure 1A); and hyperglycemia onset according fasting blood glycemia levels (Suppl. Figure 1B) and oral glucose tolerance test (Suppl. Figure 1C). Furthermore, HFD mice displayed a reduction into pancreas of phosphorylated Akt (pAkt; Suppl. Figure 1D), a recognized molecular sign of hyperglycemia [58], and decreased levels of fasting circulating Glucagon-Like Peptide 1 (GLP1) (Suppl. Figure 1E), as observed in diabetic and obese patients as well as in dysmetabolic mice [59–61]. Taken together, these pieces of evidence demonstrated the dysmetabolism onset in the HFD group already at 10 weeks of dietary regimen, time that was chosen for injection of KPC organoids into pancreata (Suppl. Figure 1A). Subsequently, dietary regimen was maintained for the entire duration of the experiments and ultrasound imaging was performed to monitor tumor formation and growth in both experimental groups. Interestingly, it was registered an organoid engraftment up to 85% independently from dietary regimen (Suppl. Figure 2A; n = 15 for each experimental group), but a statistically significant increase of the lesion volume in HFD group at two different time points (T1 = 30 days, n = 12; T2 = 90 days from orthotopic transplantation, n = 9) compared to LFD mice (T1: n = 12; T2: n = 10) (Fig. 1A). Histological characterization confirmed that lesions observed in HFD mice were representative of more advanced tumor stages in comparison to LFD mice (Suppl. Figure 2B-C) and associated with a specific transcriptomic landscape of neoplastic lesions (Fig. 1B-E and Suppl. Table 5,6). Interestingly, after pairwise comparison of HFD/LFD at T1 (Fig. 1B and Suppl. Table 5) and T2 (Fig. 1D and Suppl. Table 6), 527 and 466 genes were found respectively differentially expressed at more than ± 1 log2 fold change with a FDR < 0.05, demonstrating that HFD-associated metabolic alterations induce significant changes in the pancreatic transcriptome. To investigate the role of HFD-dependent transcripts during pancreatic cancer development, gene-ontology (GO) analysis was performed on significantly up- or down-regulated transcripts (Fig. 1C and Fig. 1E). The interconnections among the up-regulated transcripts indicated that these genes belonged to biological processes prevalently associated with collagen deposition (GO:0030199; GO:0032964; GO:0032963; GO:0038063), angiogenesis (GO:0001525; GO:0048010; GO:00038084), cell proliferation (GO:0008284; GO:0000165; GO:0048146; GO:0043410; GO:00070374), and cell migration (GO:0010718; GO:0007229; GO:0030335; GO:0034446; GO:0010763; GO:0014911) (Fig. 1C and Fig. 1E). In the same condition, the most down-regulated genes were enriched in transcripts associated with glucose homeostasis (GO:0001678), oxidative phosphorylation (GO:0022904; GO:0032981), immune response (GO:0001916; GO:0045087; GO:00002376), and gastrointestinal epithelium maintenance (GO: 0030277; GO:0003382) (Fig. 1C and E). Altogether, these results point to HFD as a molecular player involved in PDAC tumorigenesis affecting stroma deposition, altering cell metabolism and modulating immune response. Intriguingly, bioinformatics analysis of HFD/LFD comparison further reinforced the indication that HFD accelerates PDAC tumorigenesis, acting on collagen biosynthesis, fibroblast proliferation and immunomodulation (Fig. 1C and E). The HFD-associated transcriptome was further confirmed by specific analysis of collagen deposition and lymphocyte infiltration into pancreatic neoplastic lesions. Specifically, quantitative assessment of picrosirius red staining, specific for collagen network analysis [62], showed higher collagen deposition into HFD tumor pancreata in stage-matches cryosection for LFD and HFD (Fig. 1F), paralleled by a higher expression of collagen gene expression levels, as assessed by quantitative real time PCR (Fig. 1G). Furthermore, confocal analysis of stage-matched pancreatic lesions revealed a decreased number of infiltrated T lymphocytes (CD3 positive cells) into pancreatic lesions of HFD mice compared to LFD ones (Fig. 1H). These analyses point out the role of metabolic alteration in PDAC onset and progression, exacerbating two main features of tumorigenesis: higher stroma deposition and T lymphocyte exclusion.

### The higher collagen deposition observed in HFD mice associates with higher levels of P4HA1 expression and activity in PDAC stroma

**Fig. 2:**
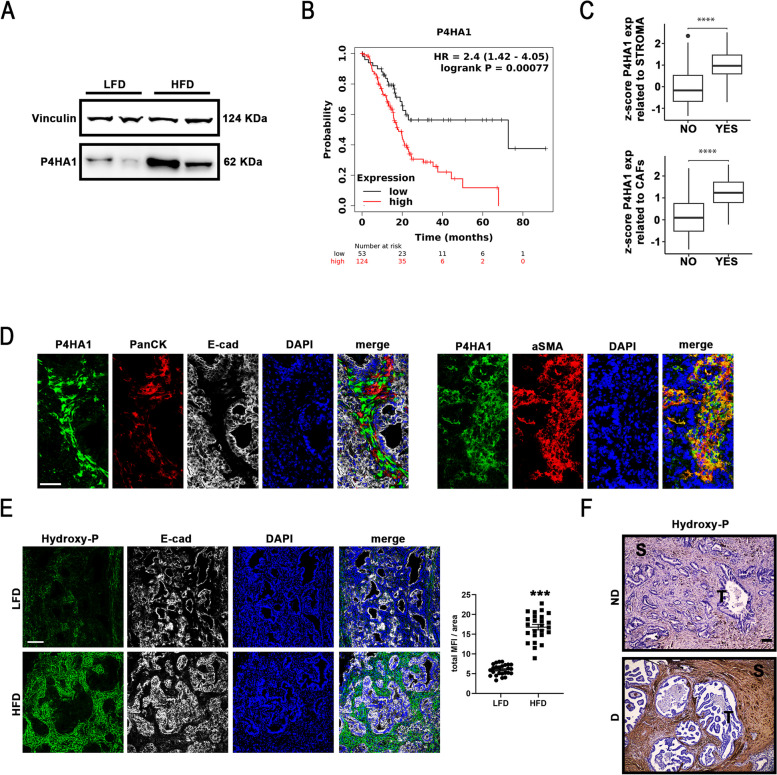
HFD mice showed a higher expression of P4HA1, a PDAC unfavorable prognostic marker, and consequent higher hydroxyproline levels. **A** Representative WB analysis of P4HA1 in LFD or HFD mice at time point T2. Loading control: vinculin (n = 4 for each group). **B** Estimation of prognostic P4HA1 in PDAC patients by Kaplan–Meier survival curves according to P4HA1 gene expression levels (low expression Vs high expression) derived from transcriptomics data deposited in TCGA repository. **C** P4HA1 levels in different PDAC stratification groups. Upper panel: transcript levels of P4HA1 (expressed as z-score) in PDAC patient data extracted from the CPTAC portal displaying significant enrichment in stromal activated signature. Lower panel: P4HA1 transcript levels (expressed as z-score) in PDAC patient data extracted from the CPTAC portal displaying significant enrichment in hCAF infiltration signature. **D** Left panels: Representative confocal microscopy images depicting HFD mouse cryosections at time point T2 probed by an anti-P4HA1 antibody (green), an anti-Pancytokeratin (PanCK; red), and an anti-E-cadherin (E-Cad, white). Nuclei were counterstained with DAPI (blue). Right panels: Representative confocal microscopy images depicting HFD mouse cryosections at time point T2 probed by an anti-P4HA1 antibody (green) and an anti-alpha smooth muscle actin (aSMA; red). Nuclei were counterstained with DAPI (blue). Merged fluorescence images are shown (right panels). Original scale bar, 50 μm; n = 5. **E** Left panels: Representative confocal microscopy images depicting LFD (upper panels) and HFD (lower panels) mouse cryosections at time point T2 probed by an anti-E-cadherin antibody (E-Cad; white, left) and anti-hydroxyproline antibody (green, middle left). Nuclei were counterstained with DAPI (blue, middle right). Merged fluorescence images are shown (right; merge). Original scale bar, 100 μm; n = 5 (***p < 0.001). Right panel: Right panels: quantification of HydroxyP MFI on area in LFD (black circles) and HFD (black squares) mice (n = 25; ***p < 0.001). **F** Representative immunohistochemistry images depicting ND- (upper panel) and D- (lower panels) human PDAC specimens probed by an anti-P4HA1 antibody. Nuclei were counterstained with hematoxylin. “S” stands for stroma enriched region; “T” stand for tumor area. Original scale bar, 100 μm; n = 3. Data expressed as average ± SEM. Data analyzed by Kolmogorov–Smirnov test

PDAC stroma features are strictly correlated with immune exclusion [11, 13, 14]. The conducted transcriptomics pointed out a significant statistical deregulation of genes involved in pathways associated with collagen biosynthesis, fibril organization and ECM deposition, thus harnessing stroma development during pancreatic neoplasia progression. For this reason, DEGs involved in collagen deposition and their sensitiveness to HFD-dependent metabolic alterations were specifically analysed. Among these genes, the expression of prolyl 4-hydroxylase subunit alpha 1 (P4HA1), a procollagen-proline, 2-oxoglutarate 4-dioxygenase whose enzymatic activity supports collagen synthesis [23, 63], was found statistically upregulated in HFD mice in comparison to LFD mice, independently on neoplastic progression advancement (Suppl. Figure 2D). Thus, P4HA1 resulted extremely sensitive to HFD-dependent metabolic alteration among the genes belonging to biological processes associated with increased collagen deposition in HFD mice bearing KPC organoids (Fig. 1 B-E), according to GO analysis. The role of this enzyme was further investigated in the pathogenesis of PDAC upon metabolic syndrome pressure to get insight possible molecular mechanisms underpinning PDAC higher collagen deposition and low anti-tumoral response associated with metabolic syndrome. Indeed, a higher P4HA1 protein level was confirmed in pancreata of HFD mice bearing KPC organoids by western blot analysis (Fig. 2A). Although P4HA1 is a known biomarker of poor prognosis in different malignancies [24, 25], at present its involvement in pancreatic neoplastic stroma development has been barely investigated. Indeed, Kaplan–Meier curve related to total P4HA1 expression generated exploiting PDAC transcriptomics data deposited in The Cancer Genome Atlas (TCGA) data sets showed an interesting inverse correlation of high P4HA1 expression with PDAC patient life expectancy [24, 25, 64], highlighting its prognostic value and prompting to further keep P4HA1 under the spotlight as crucial molecular player of pancreatic cancer stroma evolution (Fig. 2B). To this purpose, the Clinical Proteomic Tumor Analysis Consortium (CPTAC) data portal was interrogated for its rich proteogenomics dataset to correlate whether the increase of P4HA1 expression levels could be attributed to CAFs, a main population of PDAC stroma strongly supporting tumorigenesis. Specifically, PDAC data from 140 patients, available at the CPTAC repository, were normalized by sample-specific z-scoring, centered on the median, and a gene set enrichment analysis over the ranked proteomic data was performed stratifying PDAC patients according to their individual stromal infiltration signature [15]. Remarkably, PDAC patients with higher P4HA1 transcript levels, expressed as z-score, display a statistical significant enrichment in stroma infiltration signature [normalized enrichment score (NES) > 2 and adjusted p-value < 0.05] in comparison to PDAC patients in which P4HA1 expression levels were not modulated (Fig. 2C, upper panel). Moreover, the same approach was conducted to interrogate the CPTAC data portal on the enrichment of CAF infiltration signature [16]. Also, this analysis showed that PDAC patients with higher P4HA1 transcript levels display a statistical significant enrichment in CAF infiltration signature (NES > 2 and adjusted p-value < 0.05) in comparison to PDAC patients in which P4HA1 expression levels were not modulated (Fig. 2C, lower panel). These pieces of evidence suggest that the observed P4HA1 expression level increase in HFD mice bearing KPC organoids might be attributed predominantly to the stromal compartment and in particular to CAFs. In line with this, confocal analyses displayed a P4HA1 signal in the area of the pancreatic tumor lesion negative for the epithelial marker E-cadherin (E-Cad) and for the pancreatic epithelial tumor Pan-cytokeratin (PanCK) and a co-localization with the CAF marker alpha-smooth muscle actin (aSMA) (Fig. 2D), supporting the hypothesis of a specific P4HA1 role in the stromal compartment. To further investigate P4HA1 enzymatic activity, hydroxyproline levels were determined in cryo-sections of pancreata derived from LFD and HFD mice bearing KPC organoids. Indeed, P4HA1 catalyzes the hydroxylation of proline residues into 4-hydroxyproline at the Y position in the Glycine-X–Y motif of collagen α chains stabilizing collagen triple helix [23, 63]. Confocal analysis pointed out an enhancement of hydroxy-proline staining in regions of neoplastic lesions negative for E-cad in HFD compared to LFD mice (Fig. 2E). This observation suggests a higher P4HA1 activity in stromal compartment exposed to dysmetabolic condition. Immunohistochemistry analysis confirmed the overlapping of hydroxyproline staining with collagen 1A1 (Col1A1) signal in HFD mice, suggesting that the increased activity of P4HA1 might be localized in the area of collagen synthesis (Suppl. Figure 2E). The higher hydroxyproline levels associated to metabolic syndrome was further confirmed by immunohistochemistry analysis performed on paraffin embedded PDAC tissues derived from dysmetabolic (D) patients in comparison to non-dysmetabolic (ND) patients (Fig. 2F). Taken together these results shed light on P4HA1 function in PDAC stroma as a molecular player of stroma deposition in pancreatic cancer development upon metabolic syndrome, ultimately suggesting hydroxyproline levels as potential PDAC biomarkers.

### hpCAFs derived from dysmetabolic PDAC chemotherapy naïve patients showed higher levels of P4HA1, collagens, hydroxyproline and collagen contraction ability

**Fig. 3:**
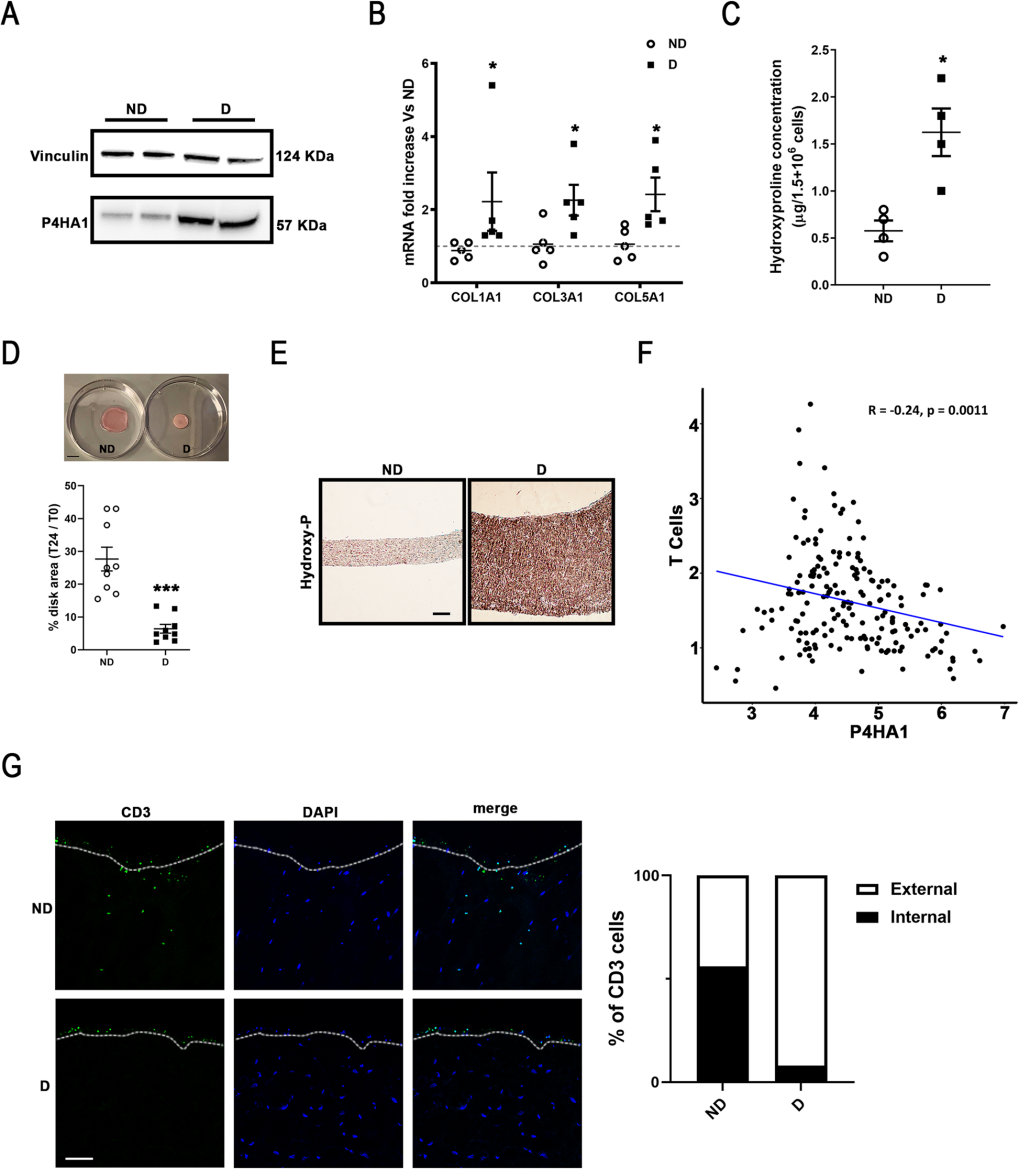
hpCAFs isolated from D PDAC patients showed higher P4HA1 protein expression paralleled by increased hydroxyproline levels and higher collagen mRNA expression contributing to higher contraction strength and immune exclusion. **A** Representative WB analysis of P4HA1 protein levels in hpCAFs isolated from ND or D PDAC patients. Loading control: vinculin (n = 5 for each group). **B** Col1A1, Col3A1, and Col5A1 mRNA analysis in hpCAFs isolated from ND (white circles) or D (black squares) PDAC patients. Data expressed as fold increase of average Ct of ND after subtraction of the housekeeping gene p0 signal (n = 4; *p < 0.05 D vs. ND patients). **C** Quantification of hydroxyproline levels in hpCAFs isolated from ND (white circles) and D (black squares) PDAC patients (n = 4 for each group; *p < 0.05 D vs. ND patients). **D** Upper panel: Representative images of contraction assay performed in hpCAFs derived from ND- or D- PDAC patients. Original scale bar, 1 cm. Lower panel: Quantification of contraction performed on hpCAFs derived from ND- (white circles) and D- (black squares) PDAC patients in cell-populated collagen hydrogel (n = 9 for each group; ***p < 0.001 D vs. ND patients). **E** Representative immunohistochemistry images depicting ND- (left panel) and D- (right panels) hpCAF-populated collagen hydrogel after 24 h contraction probed by an anti-hydroxyproline antibody. Nuclei were counterstained with hematoxylin. Original scale bar, 100 μm; n = 5. **F** Correlation plot between P4HA1 and T cell signature gene expression in PDAC patients according TCGA data showing a negative correlation coefficient (R = −0.24) with statistical significance (p = 0.001). **G** Left panels: representative confocal microscopy images depicting ND- (upper panels) and D- (lower panels) hpCAF-populated collagen hydrogel after 24 h contraction probed by an anti-CD3 antibody (green, left). Nuclei were counterstained with DAPI (blue, middle). Merged fluorescence images are shown (right; merge). Original scale bar, 100 μm; n = 5. Right panel: quantification of CD3 signal out (external white rectangle) or into (internal black rectangle) the disk. Data expressed as average ± SEM. Data analyzed by Kolmogorov–Smirnov test

To get insight into P4HA1 stromal role upon metabolic syndrome, a biobank of hpCAFs, established from 30 PDAC chemotherapy naïve patients undergoing pancreatic eco-endoscopy, was exploited (Suppl. Table 1). A specific protocol was established to isolate hpCAFs from small-size specimens obtained from eco-endoscopy samples derived from PDAC patients with (dysmetabolic, D) or without (non-dysmetabolic, ND) a history of diabetes and obesity (assumed as dysmetabolic condition). Specifically, after enzymatic digestion, a cell population with fibroblast-like morphology (Suppl. Figure 3A), more than 95% positive for mesenchymal markers, including CD29 and CD44 [65], and negative for endothelial (CD31; [66]), epithelial (EpCAM; [67]), and leucocyte (CD45; [68]) markers was isolated, expanded and bio-banked (Suppl. Figure 3B). Although, both ND- and D-hpCAFs were maintained in culture in the same condition, the characterization of their features sustains the observation that also pancreatic stromal compartment is able to retain a “metabolic memory” of dysmetabolism [21, 69]. Interestingly, higher P4HA1 protein levels were observed in D-hpCAFs compared to ND-hpCAFs (Fig. 3A), paralleled by increased mRNA levels of Col1A1, Col3A1, and Col5A1 (Fig. 3B), suggesting similar molecular alterations associated with metabolic syndrome observed in the orthotopic pancreatic cancer mouse model fed with HFD (Fig. 1 and Fig. 2). Consistently, D-hpCAFs showed higher levels of hydroxyproline concentration compared to ND-hpCAFs (Fig. 3C), further corroborating the hypothesis of P4HA1 enzymatic activity involvement in the deregulation of pancreatic stroma developing upon dysmetabolism pressure. Thereafter, a contraction assay was performed by seeding the same number of ND- and D-hpCAFs in a collagen 1 matrix, then cell-populated collagen hydrogels formed and contracted over 24 h. Subsequently, total area of cell-populated contracted hydrogel was measured in phase contrast pictures in comparison to area of hydrogel at time 0 (Fig. 3D). The contraction assay data displayed an increased ability of D-hpCAFs in cell-populated collagen hydrogel contraction (Fig. 3D). Moreover, immunohistochemistry analyses revealed a higher hydroxyproline levels in D-hpCAF populated hydrogels compared to ND-hpCAF populated ones (Fig. 3E). To test whether contraction was orchestrated by high levels of P4HA1, D-hpCAFs were silenced by two different targeted-P4HA1 knock-down strategies: siRNA and CRISPR/Cas9 approaches (Suppl. Figure 4A-F). Moreover, D-hpCAFs were also treated with a selective P4HA1 inhibitor, 1,4-DPCA (Suppl. Figure 4G and 4H) [70]. Thereafter, collagen hydrogel contraction assay was performed and the strong contraction capacity of D-hpCAFs was prevented (Suppl. Figure 4B, 4E and 4G). To further confirm P4HA1 involvement in hp-CAF populated hydrogel contraction, ND-hpCAFs, expressing low P4HA1 levels and showing less collagen contraction ability compared to D-hpCAFs, were infected with P4HA1-Myc lentiviral particles (Suppl. Figure 4I-K). Upon P4HA1 overexpression, contraction assay results showed an increased ability of P4HA1 overexpressed ND-hpCAFs in cell-populated collagen hydrogel contraction compared to Empty-vector infected ND-hpCAFs (Suppl. Figure 4J) paralleled to an increase in hydroxyproline levels (Suppl. Figure 4K). These pieces of evidence further support a role of P4HA1 in collagen fibril contraction upon dysmetabolic conditions. As a readout of P4HA1 activity, hydroxyproline levels were assessed in all loss and gain of function experiments (Suppl. Figure 4C, 4F, 4H, and 4 K). These results prompt to hypothesize that metabolic syndrome affects P4HA1 expression and ultimately stromal features of PDAC, increasing collagen contraction strength and contributing to establish an impermeable TME for therapies and immune system.

### Semaglutide treatment decreases stroma development and allows T lymphocyte infiltration in ex vivo and in vivo models of PDAC developing upon metabolic syndrome

**Fig. 4:**
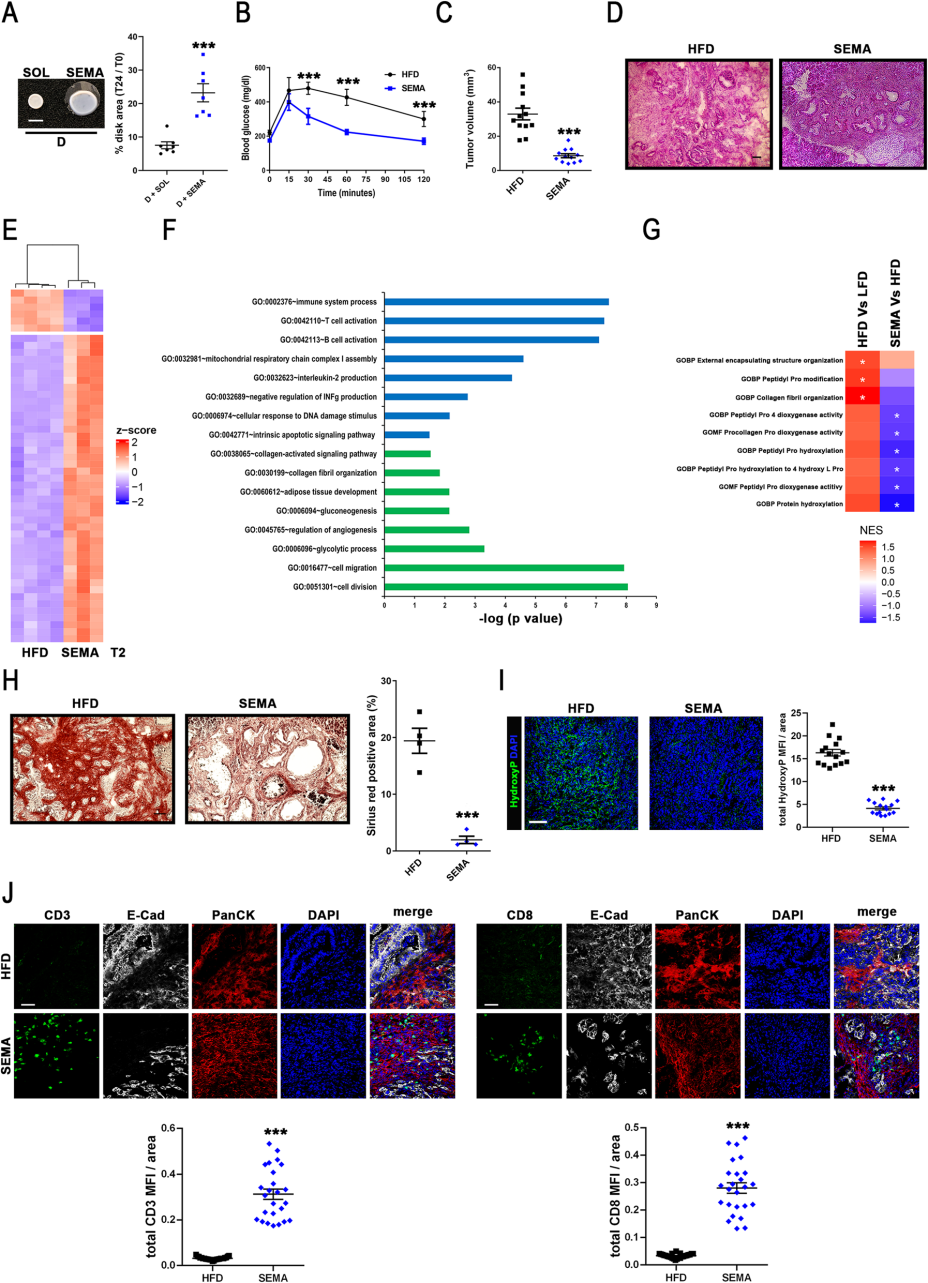
Glycemic control achieved by Semaglutide administration decreases PDAC stroma deposition favoring immune infiltration. **A** Left panel: Representative images of contraction assay performed in hpCAFs derived from D-PDAC patients untreated (SOL) or treated with SEMA. Original scale bar, 1 cm. Right panel: Quantification of contraction performed on hpCAFs derived from D-PDAC patients untreated (black cicrcles) or treated (blue squares) with SEMA in cell-populated collagen hydrogel (n = 7 for each group; ***p < 0.001 SEMA vs. SOL D-hpCAFs). **B** Oral glucose tolerance test (OGTT) in HFD (black circles) and HFD treated with semaglutide (SEMA; blue squares) mice (n = 3 for each group; ***p < 0.001 SEMA vs. HFD). **C** In vivo tumor size measurement at 60 days from treatment start in HFD (black squares; n = 12) and SEMA (blue rumbles; n = 12) mice bearing pre-neoplastic lesions (***p < 0.001 SEMA vs. HFD). **D** Representative hematoxilin/eosin images of pancreatic neoplasia at T2 time point in HFD (left panel) and SEMA (right panel) mice bearing pre-neoplastic lesions. Original scale bar, 100 μm; n = 5. **E** Heatmap showing the 50 most differentially regulated genes in HFD and SEMA mice at time point T2 identified by total RNA sequencing analysis (n = 4 for HFD group and n = 3 for SEMA group). Red and blue represent over- and under-expressed genes, respectively. **F** Gene ontology analysis of differentially regulated transcripts between HFD and SEMA mice at time point T2. Up-regulated genes depicted in blue bars and down-regulated genes in green bars. **G** Heatmap showing P4HA1 signature according GSEA analysis in T2 and SEMA mice identified by bioinformatics RNA sequencing analysis (n = 3 for SEMA group; n = 4 for LFD and HFD T2). Red and blue represent over- and under-categories, respectively. **H** Left panels: Representative picrosirius red staining images of pancreatic lesions at time point T2 in HFD (left panel) and SEMA (right panel) mice bearing pre-neoplastic lesions. Right panel: Quantitative assessment of percentage of picrosirius red stained positive area in HFD (black squares) and SEMA (blue rumbles) mice at time point T2 (n = 4 for each group; ***p < 0.001 SEMA vs. HFD). **I** Left panels: Representative immunofluorescence images depicting HFD (left panel) and SEMA (right panel) mouse cryosections at time point T2 probed by an anti-hydroxyproline antibody (green). Nuclei were counterstained with DAPI (blue). Merged images depicted. Original scale bar, 100 μm; n = 5. Right panels: quantification of hydroxyproline positive cells in HFD (black squares) and SEMA (blue rumbles) mice (n = 15). **J** Upper left panels: Representative confocal microscopy images depicting HFD (upper panels) and SEMA (lower panels) mouse cryosections at time point T2 probed by an anti-CD3 antibody (green), an anti-E-Cadherin (white), and an anti-pan cytokeratin (red). Nuclei were counterstained with DAPI (blue). Merged pictures were depicted on the right. Original scale bar, 50 μm; n = 5. Upper right panels: Representative confocal microscopy images depicting HFD (upper panels) and SEMA (lower panels) mouse cryosections at time point T2 probed by an anti-CD8 antibody (green), an anti-E-Cadherin (white), and an anti-pan cytokeratin (red). Nuclei were counterstained with DAPI (blue). Merged pictures were depicted on the right; n = 5. Left lower panel: quantification of CD3 positive cells in HFD (black squares) and SEMA (blue rumbles) mice. Right lower panels: quantification of CD8 positive cells in HFD (black squares) and SEMA (blue rumbles) mice (n = 25 for each group; ***p < 0.001 SEMA vs. HFD). Data expressed as average ± SEM. Data analyzed by Kolmogorov–Smirnov test (A, C, H, I, J) or 2-way-ANOVA (B)

To further explore the active role of P4HA1 role in stroma development, data related to P4HA1 and immune cell signature [55] gene expression levels of PDAC patients deposited in TCGA data portal were correlated, revealing that P4HA1 expression enrichment associates with decreased T lymphocyte signature expression (Fig. 3F). This statistically significant inverse correlation between P4HA1 and T lymphocyte gene expression levels and results obtained from GO analysis showing an impairment of T cell mediated cytotoxicity (GO:0001916; Fig. 1E) prompted to consider the involvement of P4HA1 in T lymphocyte exclusion from PDAC, a typical cold tumor [71, 72]. To further analyze P4HA1 role in T lymphocyte exclusion, collagen hydrogel contraction assay was performed in the presence of human purified T lymphocytes. Specifically, the same number of ND- and D-hpCAFs was seeded in a collagen 1 matrix, cell-populated collagen hydrogels formed and contracted over 24 h, thereafter 2 × 10^6^ human T lymphocytes were seeded on the top of contracted hydrogel and left overnight to allow their infiltration into the hydrogel. Confocal analysis revealed that human T lymphocytes (CD3 + cells) were able to penetrate ND-hpCAF collagen hydrogels, whereas D-hpCAF collagen hydrogels resulted impermeable to them (Fig. 3G), suggesting that the higher D-hpCAF-dependent hydrogel contraction might contribute to T lymphocyte exclusion. Since P4HA1 expression resulted sensitive to dysmetabolism condition (Suppl. Figure 2D), a treatment able to counteract metabolic syndrome was tested in an attempt to prevent stroma development observed in dysmetabolic patients. Specifically, Semaglutide (SEMA), a glucagon-like peptide-1 receptor agonist (GLP-1 RA) molecule approved for the treatment of both type 2 diabetes (T2D) and obesity associated with significant improvement in metabolic syndrome parameters [43, 44], was tested since fasting HFD mice showed lower GLP-1 circulating levels (Suppl. Figure 1E) compared to fasting LFD mice and D-hpCAFs express GLP-1R protein according WB analysis (Suppl. Figure 5A). Moreover, upon SEMA treatment D-hpCAFs displayed decreased Col1A1 and P4HA1 expression levels (Suppl. Figure 5B), suggesting SEMA as a promising therapeutic intervention to partially revert dysmetabolic dependent pCAF alterations. Besides, contraction assay was performed on D-hpCAFs previously treated or not for 24 h with 240 nmol SEMA. Noteworthy, SEMA treatment was able to rescue contraction strength of D-hpCAF populated collagen hydrogels (Fig. 4A), further supporting an interesting potential therapeutic effect of SEMA in decreasing pancreatic stromal deposition, ultimately improving anti-tumor immune response. To test this hypothesis, a subgroup of HFD mice, randomly chosen 1 week before PDAC organoid engraftment, were treated with 30 nmol/kg SEMA (SEMA mice; n = 18) for 6 weeks. During treatment, metabolic parameters were monitored. As expected, SEMA mice showed lower levels of body weight gaining (Suppl. Figure 5C) and fasting blood glycemia levels (Fig. 4B). Intriguingly, although SEMA did not affect pre-neoplastic lesion organoid engraftment (Suppl. Figure 5D), a statistically significant decreased tumor volume was observed in SEMA mice (Fig. 4C), paralleled by a less advanced tumor staging compared to HFD mice as revealed by immuno-histochemistry analysis (Fig. 4D and Suppl. Figure 5E). These pieces of evidence further corroborate the therapeutic potential of SEMA in pancreatic cancer. To deepen characterize SEMA role during pancreatic cancer development, a transcriptomic analysis was performed. Noteworthy, after pairwise comparison of SEMA/HFD at T2 (Fig. 4E and Suppl. Table 7), 2361 genes were found differentially expressed at more than ± 1 log2 fold change with an FDR < 0.05, demonstrating that SEMA treatment induces significant changes in the transcriptome of pancreatic lesions. GO analysis on significantly up- or down-regulated transcripts supported the ability of SEMA to counteract stroma development improving anti-tumoral response (Fig. 4F). Indeed, the interconnections among the up-regulated transcripts indicated that these genes belonged to biological processes prevalently associated with immune response (GO:0002576; GO:0042110; GO:0042113; Fig. 4F). In the same condition, the most down-regulated genes were enriched in transcripts associated with collagen deposition (GO:0038045; GO:0030100), adipose tissue development (GO:0060512); glycolytic process (GO:0059096), cell migration (GO:00164777), and cell division (GO:00551301; Fig. 4F). Altogether, these results point to SEMA as a potential therapeutic option for PDAC developing in patients affected by metabolic syndrome mostly influencing stroma deposition and modulating immune response, thus re-shaping desmoplasia. Intriguingly, bioinformatics analysis of SEMA/HFD comparison further reinforced the indication that SEMA might impinge pCAF functions in collagen biosynthesis with immunomodulation properties. Indeed, differentially regulated transcripts belonging to GO biological processes involved in stroma deposition and immune response in SEMA, HFD and LFD mice bearing KPC organoids clearly showed the therapeutic potential of this anti-dysmetabolic treatment in PDAC (Suppl. Figure 4F and Suppl. Table 8). GO analysis results prompted to perform a network-based deconvolution approach in an attempt to profile tumor microenvironment composition starting from bulk RNAseq data. Specifically, a T lymphocyte signature [52] and a pancreatic mesenchymal stroma cell signature [53] were exploited to estimate T cell infiltration levels and stroma cell enrichment, respectively. Interestingly, mesenchymal stroma cell signature was found decreased in SEMA group (Suppl. Figure 5F), whereas T lymphocyte cell signature was found enriched in comparison to HFD mice (Suppl. Figure 5G). These obtained results support conclusion to a role of SEMA on stroma enrichment decrease paralleled to T lymphocyte infiltration increase. Moreover, the gene set enrichment analysis (GSEA) of the P4HA1 signature of HFD/LFD and SEMA/HFD comparisons displayed a statistical significant difference in NES related to peptidyl proline dioxygenase activity, in protein hydroxylation and in collagen fibril organization corroborating SEMA role in counteracting stroma development (Fig. 4G). To further validate SEMA activity, analysis of collagen deposition, hydroxyproline level quantification and CD8 + T lymphocyte infiltration evaluation was performed in stage-matched cryosections derived from HFD and SEMA mice. Specifically, quantitative assessment of picrosirius red staining displayed decreased levels of collagen deposition into SEMA tumor pancreata compared to HFD mice (Fig. 4H), paralleled by reduced levels of hydroxyproline levels (Fig. 4I and Suppl. Figure 5I). Furthermore, confocal analysis of pancreatic lesions revealed an increased number of T lymphocyte (CD3 + cells) into the pancreatic lesions of SEMA mice compared to HFD mice with a CD8 + T cell phenotype (Fig. 4J). Altogether these results pave the way to novel therapeutic options for PDAC patients affected by metabolic syndrome, exploiting a treatment able to counteract at the same time metabolic derangements and PDAC stroma features possibly allowing immune infiltration.

## Discussion

These results oriented to the characterization of PDAC stroma development upon dysmetabolic conditions took advantage of a preclinical PDAC model, able to recapitulate the parallel development of pancreatic pre-neoplastic lesions and the surrounded resident stroma [56]; and a biobank of hpCAFs derived from chemotherapy naïve PDAC patients with or without a history of metabolic syndrome. The preclinical PDAC model adopted shows a slower PDAC development starting from synchronic pre-neoplastic lesions, offering the possibility to dissect how dysmetabolism affects PDAC stroma features. The exploited pancreatic pre-neoplastic lesion organoid transplantion in syngeneic mouse model resulted a good approximation of human PDAC pathology, which develops slowly together with desmoplasia establishment [46, 56, 57]. Moreover, since at the time of diagnosis, 50% of patients presents metastases, 29% local/regional spread, and only 3% pancreatic carcinoma in situ, less than 15% of patients are eligible for surgical resection, compromising research efforts for inadequate number of available human samples [9–11, 13, 14]. The possibility to establish an hpCAF biobank from a cohort of PDAC patients undergoing pancreatic echo-endoscopy gave the opportunity to have a valuable source of human specimens for hpCAF isolation to conduct studies aimed at establishing dysmetabolism role specifically on pancreatic tumor stroma.

Metabolic syndrome associates with a higher PDAC risk [4, 6, 8, 36]. Indeed, different meta-analysis studies pointed out the correlation of obesity and hyperglycemia with increased PDAC incidence [7, 8, 36]. Nevertheless, the molecular mechanisms linking metabolic syndrome to PDAC are still under extensive investigation and at moment the specific role of metabolic alterations on the stroma compartment has been barely investigated. This complexity is reflected in the many possible ways in which metabolic syndrome might influence high desmoplasia deposition of PDAC, resulting in a dramatic increase in disease aggressiveness, poorer response to treatments, and a decrease in overall patient survival. The pro-fibrotic role of hyperglycemia and obesity has been already demonstrated in liver and heart [73, 74], but at moment no dedicated scientific reports analysed their involvement in collagen deposition during PDAC stroma development, although a higher malignant stroma has been observed in dysmetabolism-dependent pancreatic cancer [7, 8, 36, 75–77].

Moreover, even if P4HA1 has been indicated as a prognostic biomarker of several cancers [24, 25, 64], at present no dedicated studies have analysed its sensitivity to TME developed upon metabolic syndrome. The contribution of P4HA1 in tumor progression concerns the ability to stimulate migration, invasion and angiogenesis and to support cancer cell dedifferentiation during tumoral progression, when aggressive tumors develop following epithelial-mesenchymal transition activation [26–32]. P4HA1 pivotal role in collagen biosynthesis and fibril formation is well characterized [23, 63], but the link among metabolic syndrome, ECM deposition and PDAC stroma, objective of the present research work, assumes unprecedented features exploitable for diagnostic and therapeutic purposes in PDAC clinical practice. In addition to this, P4HA1 immune-modulatory role in cancer has been only recently analysed in different cancer types where its increase associated with worse cancer prognosis and lower overall survival, correlates with an immunosuppressive TME and reduced anti-tumoral response [24, 25, 64]. However, most of these researches based their pieces of evidence on mathematical modelling studies derived from cancer genome data portals [24, 25, 78, 79]. These results supports data obtained in the present research work, where P4HA1 immuno-modulatory role has been demonstrated associated with a higher stroma deposition of dysmetabolic-dependent PDAC desmoplasia by wet laboratory experiments (Fig. 2 and Fig. 3), thus linking its well-known enzymatic activity in collagen biosynthesis to establish a physical barrier preventing T lymphocyte infiltration and ultimately anti-tumoral response in PDAC, one of the cold tumor by definition [71, 72]. The present research work focused on the interplay between high P4HA1 expression levels and low T lymphocyte infiltration in PDAC developed upon dysmetabolism according to RNASeq and correlation results. Further research effort needs to show how P4HA1 increased expression level could affect other cell types involved in the anti-tumoral response. Data obtained depicted an increased expression of P4HA1 in hpCAFs (Fig. 3A), TME residing cells responsible among the other functions of ECM deposition and particularly sensitive to dysmetabolism [17–21, 69]. As previously demonstrated, cells of the stroma compartment achieve specific features upon dysmetabolic conditions, which are retained also in the presence of anti-dysmetabolic medicaments, demonstrating that they can retain the so called “metabolic memory” and resulting particularly sensitive to metabolic alterations [21, 69]. Here, it has been shown that isolated D-hpCAFs retain memory of dysmetabolism increasing P4HA1 expression and consequently its enzymatic function, as demonstrated by hydroxyproline increased levels (Fig. 3C), ultimately enhancing collagen deposition and contraction (Fig. 3B and D), thus stroma development, which might explain the higher PDAC aggressiveness in dysmetabolic patients partly due to absence of tumor T lymphocyte infiltration.

In this light, a drug, aimed to counteract metabolic syndrome clinical manifestations, was tested to verify whether it might contribute to reshape PDAC stroma improving prognosis. Indeed, it has been demonstrated that therapeutic approaches aimed at PDAC stroma depletion were inefficacious, prompting to look for strategies able to reprogram PDAC stroma [72]. Here, it has been demonstrated for the first time that SEMA, a GLP-1RA with known pharmacological effects on metabolic syndrome [43, 44], is able to reshape PDAC stroma preventing T lymphocyte exclusion by P4HA1-collagen-pCAF axis (Fig. 3 and Fig. 4). The observation of GLP-1R expression on hp-CAFs (Suppl. Figure 5A) further corroborates the direct effect of SEMA on PDAC stroma. Instead, other anti-diabetic agents not belonging to GLP-1RA class, including metformin, might only have an indirect effect on GLP-1 pathway and on PDAC stroma preventing cancer development through different mechanisms, mainly harnessing PDAC cancer cells [80]. P4HA1 expression decrease was already observed in response to another GLP-1RA, the liraglutide, in a completely different pathological context [74]. Specifically, authors showed that liraglutide was able to reduce myocardial fibrosis, acting on cardiac fibroblasts exposed to high glucose, targeting P4HA1 expression [74]. The observed immunomodulatory effect of SEMA in PDAC could benefit of further investigation exploiting mouse strains lacking T cells. However, very recently Stanisavljevic et al. demonstrated the effect of SEMA on breast cancer [81]. Specifically, upon treatment they observed a breast cancer progression deceleration as consequence of an acquired antitumor immunity boost [81]. In this light, the observations object of the present work underline SEMA therapeutic potential and pave the way for novel therapeutic protocols in combination with conventional chemotherapy and immune checkpoint inhibitors in an attempt to counteract PDAC aggressiveness in patients affected by metabolic syndrome or showing higher hydroxyproline levels.

## Conclusions

The present work elucidate mechanisms that associate metabolic syndrome with a higher PDAC aggressiveness. According to obtained results, this association can be explained by a more hostile stroma, which upon dysmetabolism additional enhances ECM deposition, enriching collagen biosynthesis and further reducing the already barely detectable immune infiltration in PDAC. The underlying molecular mechanism allowing this dysmetabolic dependent effect involves P4HA1 function, an enzyme deputed to collagen biosynthesis and fibril formation [23, 63], in the stromal compartment, putting it under the spotlight as a potential early PDAC biomarker particularly sensitive to metabolic alterations. Indeed, P4HA1 activity increase in hpCAFs boosts hydroxy-proline levels (Fig. 3A-C) and consequent PDAC stromal development intensifying collagen contraction strength, thus creating impermeable TME to both therapies and immune cell infiltration. Interestingly, SEMA, a GLP-1RA able to counteract two clinical conditions describing metabolic syndrome, hyperglycemia and obesity, is able to prevent the higher stromal development of PDAC observed in dysmetabolic conditions (Fig. 4H). Specifically, SEMA by altering stroma deposition allows T lymphocyte infiltration (Fig. 4J), possibly emerging as a further therapeutic option for PDAC patients affected by metabolic syndrome with the ability to reshape pancreatic TME.

## Supplementary Information

Supplementary Material 1. Figures S1–S5 and Tables S1-S8.

## Data Availability

The RNA sequencing datasets are publicly available at NCBI’s Gene Expression Omnibus (GEO) repository, under accession number GSE266899 located at https://www.ncbi.nlm.nih.gov/geo/query/acc.cgi?acc=GSE266899. Any additional information required to reanalyze the data reported in this work paper is available from the lead contact upon request. Further information and requests for resources should be directed to and will be fulfilled by the lead contact, Dr. Francesco Spallotta (francesco.spallotta@uniroma1.it). This study did not generate new unique reagents.

## Abbreviations

GI: Gastrointestinal
PDAC: Pancreatic ductal adenocarcinoma
ECM: Extracellular matrix
hpCAFs: Human pancreatic cancer associated fibroblasts
TME: Tumor microenvironment
LFD: Low fat diet
HFD: High fat diet
OGTT: Oral glucose tolerance test
H&E: Hematoxilin/eosin
PanIN: Pancreatic intraepithelial neoplasia
NGS: Next generation sequencing
SEMA: Semaglutide
GO: Gene ontology
NES: Normalized enrichment score
CPTAC: Clinical proteomic tumor analysis consortium
ND: Non-dysmetabolic
D: Dysmetabolic
TCGA: The cancer genome atlas
GLP1: Glucagon like peptide 1
GLP1-RA: Glucagon like peptide 1 receptor agonist
DEGs: Differentially expressed genes
P4HA1: Prolyl 4-hydroxylase subunit alpha 1
E-cad: E-cadherin
PanCK: Pancytokeratin
aSMA: Alpha smooth muscle actin
T2D: Type 2 diabetes
GSEA: Gene Set Enrichment Analysis
