# Supplementary material for: The GLP-1R agonist semaglutide reshapes pancreatic cancer associated fibroblasts reducing collagen proline hydroxylation and favoring T lymphocyte infiltration
Source: J Exp Clin Cancer Res. 2025 Jan 20;44:18. doi: 10.1186/s13046-024-03263-w (PMC11744909; doi:10.1186/s13046-024-03263-w)
Supplement: Supplementary file 1 — Supplementary Material 1. Figures S1–S5 and Tables S1-S8. [file 13046_2024_3263_MOESM1_ESM.pdf]

Supplementary figures and tables.

Suppl. Fig. 1

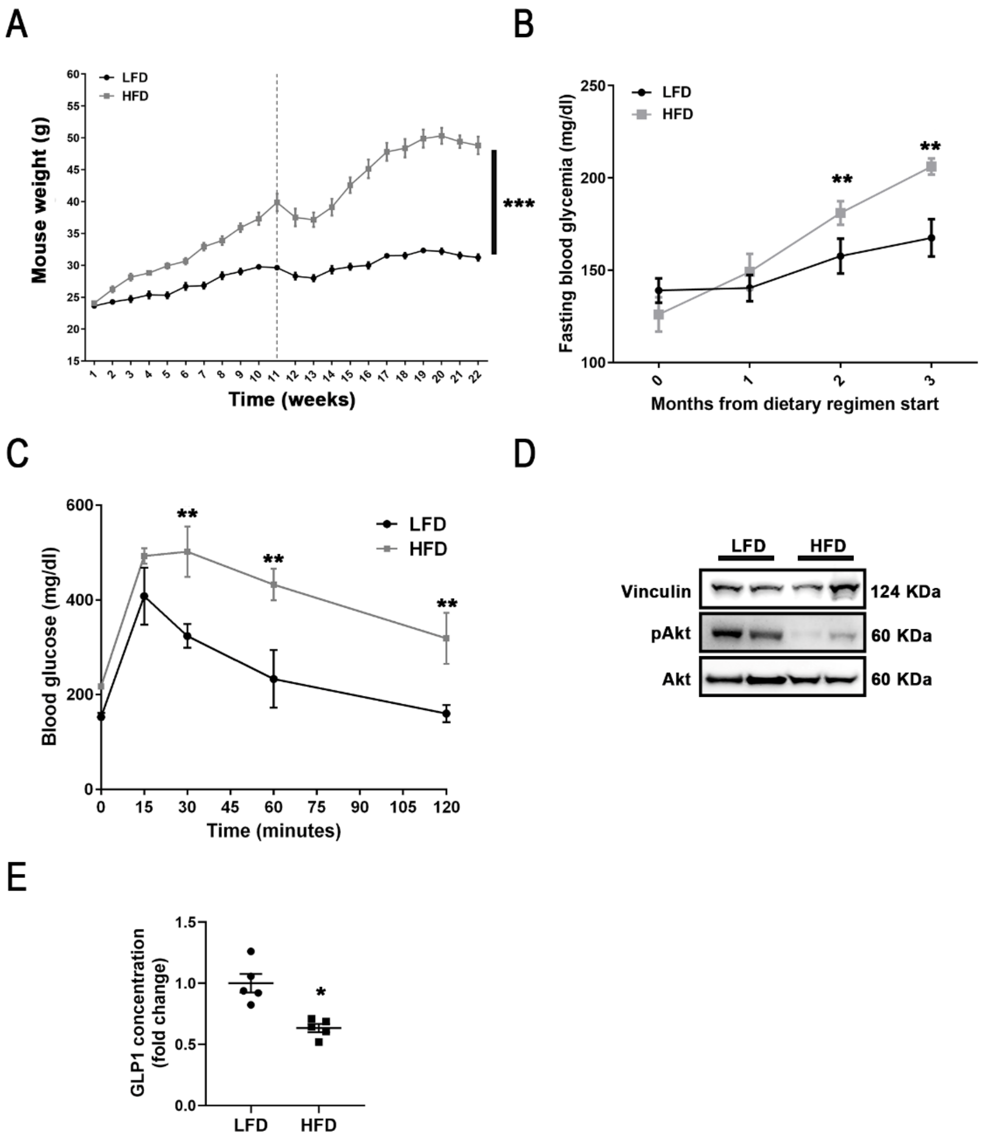

Suppl. Fig. 1 Metabolic and biochemical parameter evaluation in LFD and HFD mice. A. Animal body weight during low fat diet (LFD; black circles) and high fat diet (HFD; grey squares) feeding. Dashed line indicates when injection into pancreata of pre-neoplastic lesions derived from pancreatic organoids of KPC mice occurred (n = 18 for each group; ***p<0.001). **B.** Fasting blood glucose quantification in LFD (black circles) and HFD (grey squares) mice after 1, 2, and 3 months from dietary regimen start (n = 6 for each group; **p<0.01). **C.** Oral glucose tolerance test (OGTT) in LFD (black circles) and HFD (grey squares) mice (n = 3 for each group; **p<0.01). **D.** Representative WB analysis of pAKT, and total AKT in pancreata of LFD or HFD mice at 2 months from dietary regimen start. Loading control: vinculin (n = 4 for each group). **E.** Quantification of fasting GLP1 plasma levels in LFD (black circles) and HFD (black squares) mice (n = 5 for each group; *p < 0.05 HFD vs. LFD

mice). Data expressed as average±SEM. Data analyzed by 2-way ANOVA (A, B,C) or by Kolmogorov–Smirnov test (E).

Suppl. Fig. 2

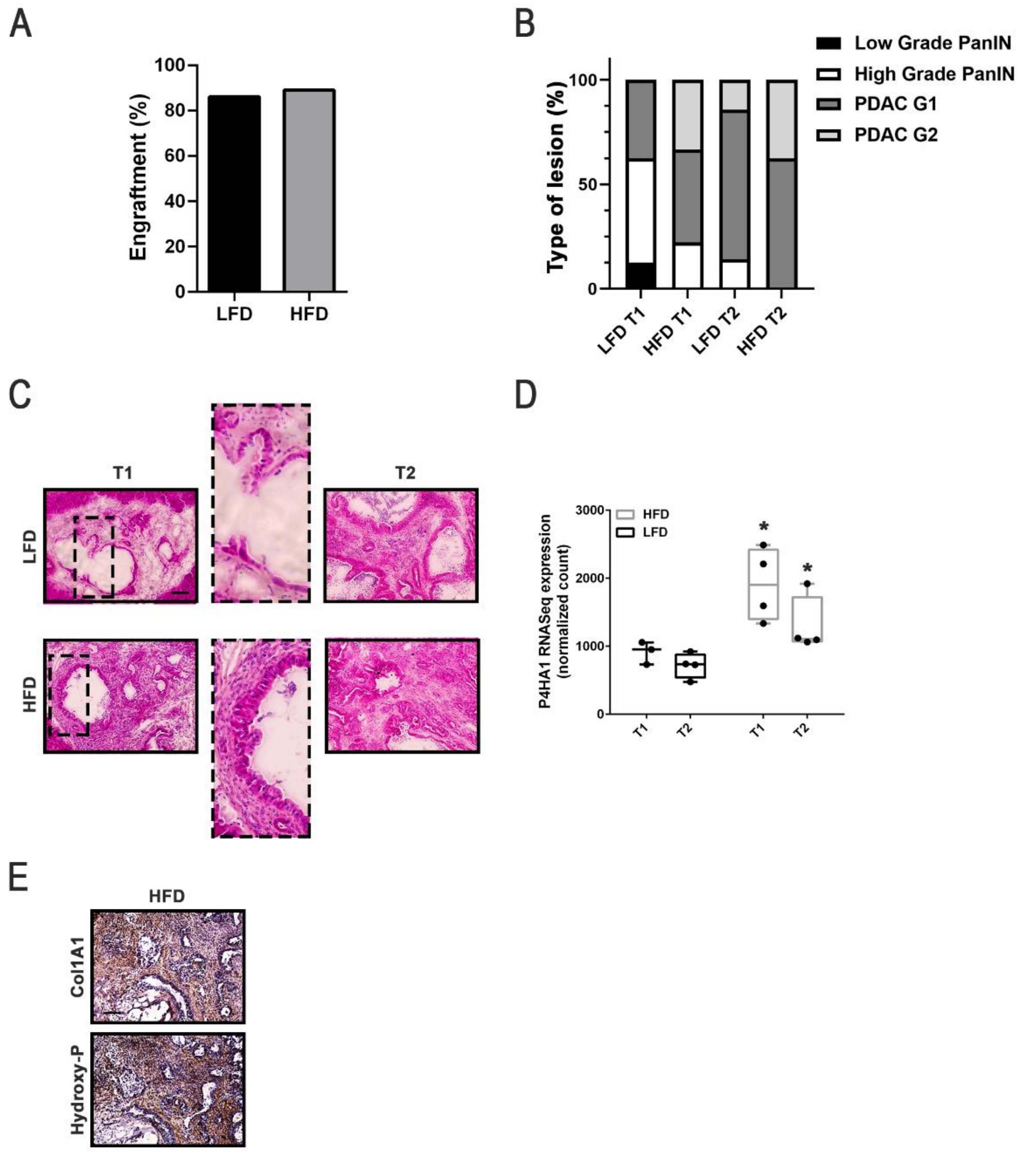

Suppl. Fig. 2 Pancreatic cancer development evaluation in LFD and HFD mice bearing pre-neoplastic lesions derived from pancreatic organoids of KPC mice. A. KPC organoid engraftment evaluation in LFD and HFD mice after a month from injection into pancreata (n= 15 for each group). **B.** Tumor score of pancreatic cancer progression quantification assigned by a pathologist specialized in pancreatic cancer histology blinded to treatment (n= 15 for each group). **C.** Representative hematoxilin/eosin images of pancreatic neoplasia at time point T1 (left panels) and T2 (right panels) in LFD (upper panels) and HFD (lower panel) mice bearing pre-neoplastic lesions (n= 15 for each group). Middle panels depict inset enlargement of T1 pictures. Original scale bar, 100 µm. **D.** P4HA1 expression levels indicated as normalized count derived from RNASeq analysis in LFD and HFD mice at T1 and T2 (n=4 for each experimental group expect for LFD T1, n=3; *p<0.05). **E.** Representative immunohistochemistry images depicting HFD mouse cryosections at time point T2 probed by

an anti-Col1A1 antibody (left panels) and anti-hydroxyproline antibody (right panels). Nuclei were counterstained with hematoxylin. Original scale bar, 100 μm; n = 5.

Suppl. Fig. 3

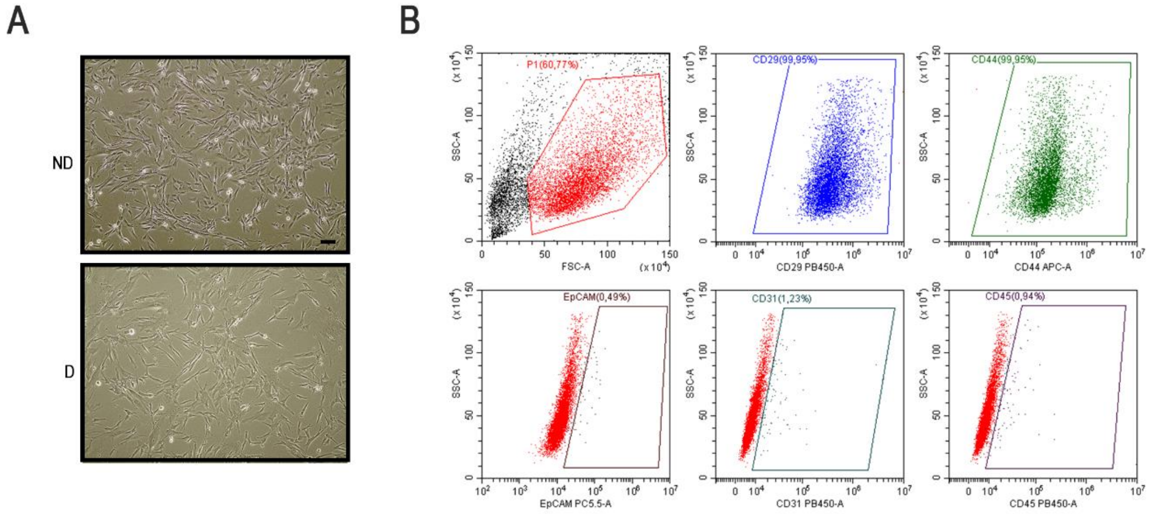

Suppl Fig 3. Phenotypic characteristics of hpCAFs isolated from PDAC patient specimens. A. Representative phase contrast images of hpCAFs isolated from non-dysmetabolic (ND; upper panel; n = 14) or dysmetabolic (D; lower panel; n = 16) PDAC patients. Original scale bar, 100 μm. **B.** Fluorescence activated cell sorter analysis of hpCAFs isolated from PDAC patients (ND: n = 14; D: n = 16). Upper left cytogram: representative scatter plot showing the forward (FSC-A) and side scatter (SSC-A) distribution of the hpCAF population. Middle upper panels: representative dot plot showing the pacific blue (PB) and SSC-A distribution of the hCAF population in the presence of CD29 antibody . Upper right cytogram: representative dot plot showing the allophycocyanin (APC) and SSC-A distribution of the hpCAF population in the presence of CD44 antibody. Lower left cytogram: representative dot plot showing the cyanine-5.5 (P5.5) and SSC-A distribution of the hCAF population in the presence of EpCAM antibody. Middle lower cytogram: representative dot plot showing the PB and SSC-A distribution of the hCAF population in the presence of CD31 antibody. Lower right cytogram: representative dot plot showing the PB and SSC-A distribution of the hpCAF population in the presence of CD45 antibody. Gating strategy and identification of hpCAFs: within total acquired events, first the debris (low FCS) was excluded (top left), followed by IgG control antibody signal.

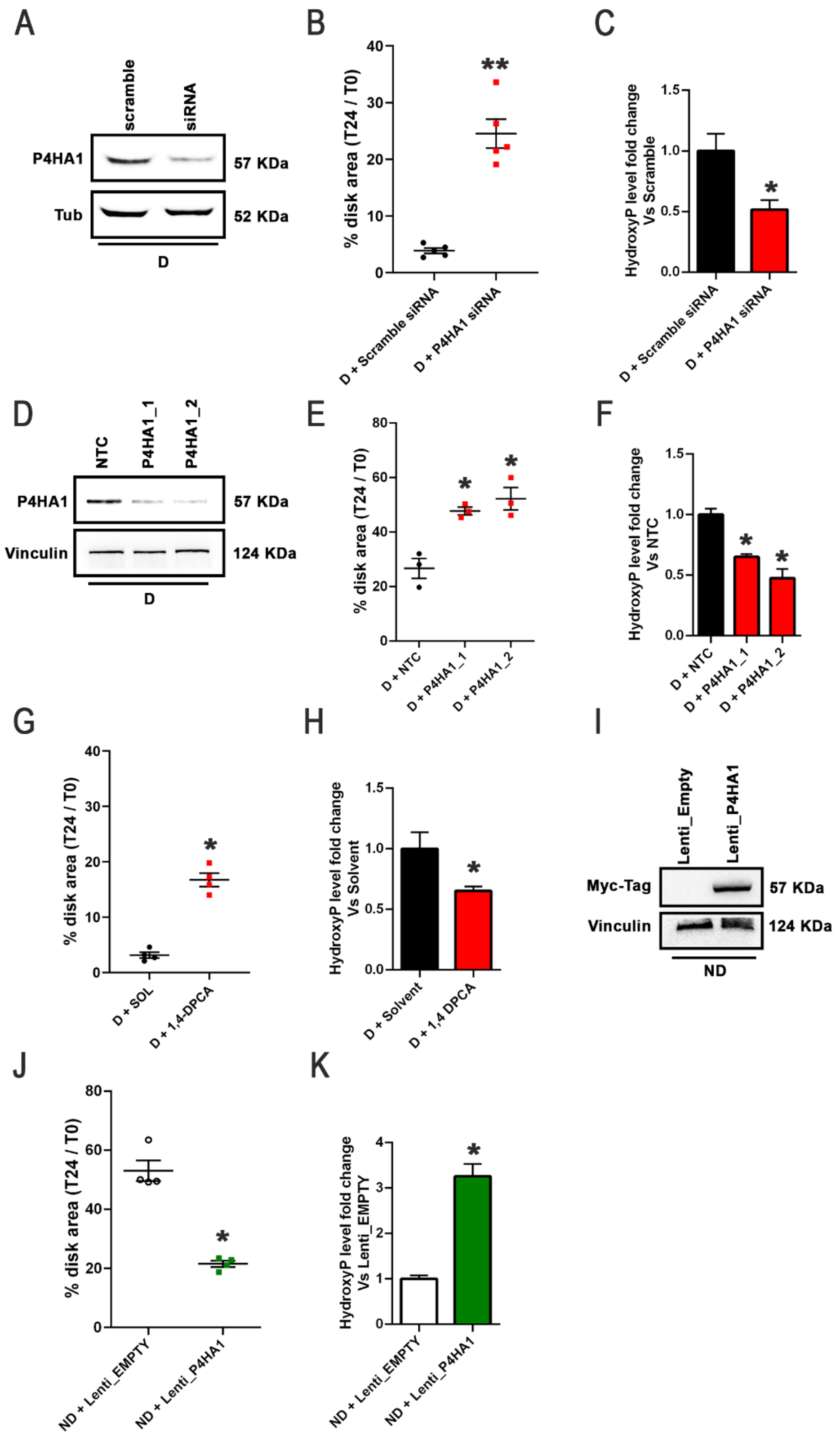

Suppl Fig 4. P4HA1 loss and gain of function in hpCAFs. A. Representative WB analysis of P4HA1 in D-hpCAFs treated with scramble or P4HA1 siRNA. Loading control: tubulin (n = 5 for each group). **B.** Quantification of contraction performed on D-hpCAFs depleted or not with a specific P4HA1 siRNA, in cell-populated collagen

hydrogel (n = 5 for each group; **p<0.01). **C.** Quantification of hydroxyproline levels in scramble or P4HA1 siRNA D-hpCAF-populated collagen hydrogel (n =3 for each group; *p<0.05). **D.** Representative WB analysis of P4HA1 in D-hpCAFs electroporated with specific human P4HA1 sgRNAs (P4HA1_1 and P4HA1_2) or non-targeting control (NTC) sgRNA and SpCas9 RNA. Loading control: vinculin (n = 4 for each group). **E.** Quantification of contraction performed on D-hpCAFs depleted or not with a specific P4HA1 sgRNA, in cell-populated collagen hydrogel (n = 3; *p<0.05). **F.** Quantification of hydroxyproline levels in NTC, P4HA1_1 or P4HA1_2 sgRNA D-hpCAF-populated collagen hydrogel (n =3 for each group; *p<0.05). **G.** Quantification of contraction performed on D-hpCAFs treated or not with a specific P4HA1 inhibitor, 1,4-DPCA, in cell-populated collagen hydrogel (*p<0.05). **H.** Quantification of hydroxyproline levels in 1,4-DPCA treated or not D-hpCAF-populated collagen hydrogel (n =3 for each group; *p<0.05). **I.** Representative WB analysis of P4HA1 in ND-hpCAFs infected with Lenti_EMPTY or Lenti_P4HA1. Loading control: vinculin (n = 4 for each group). **J.** Quantification of contraction performed on P4HA1 overexpressed ND-hpCAFs in cell-populated collagen hydrogel (n = 3; *p<0.05). **K.** Quantification of hydroxyproline levels in P4HA1 overexpressed or not ND-hpCAF-populated collagen hydrogel (n =3 for each group; *p<0.05). Data expressed as average±SEM. Data analyzed by Kolmogorov–Smirnov test.

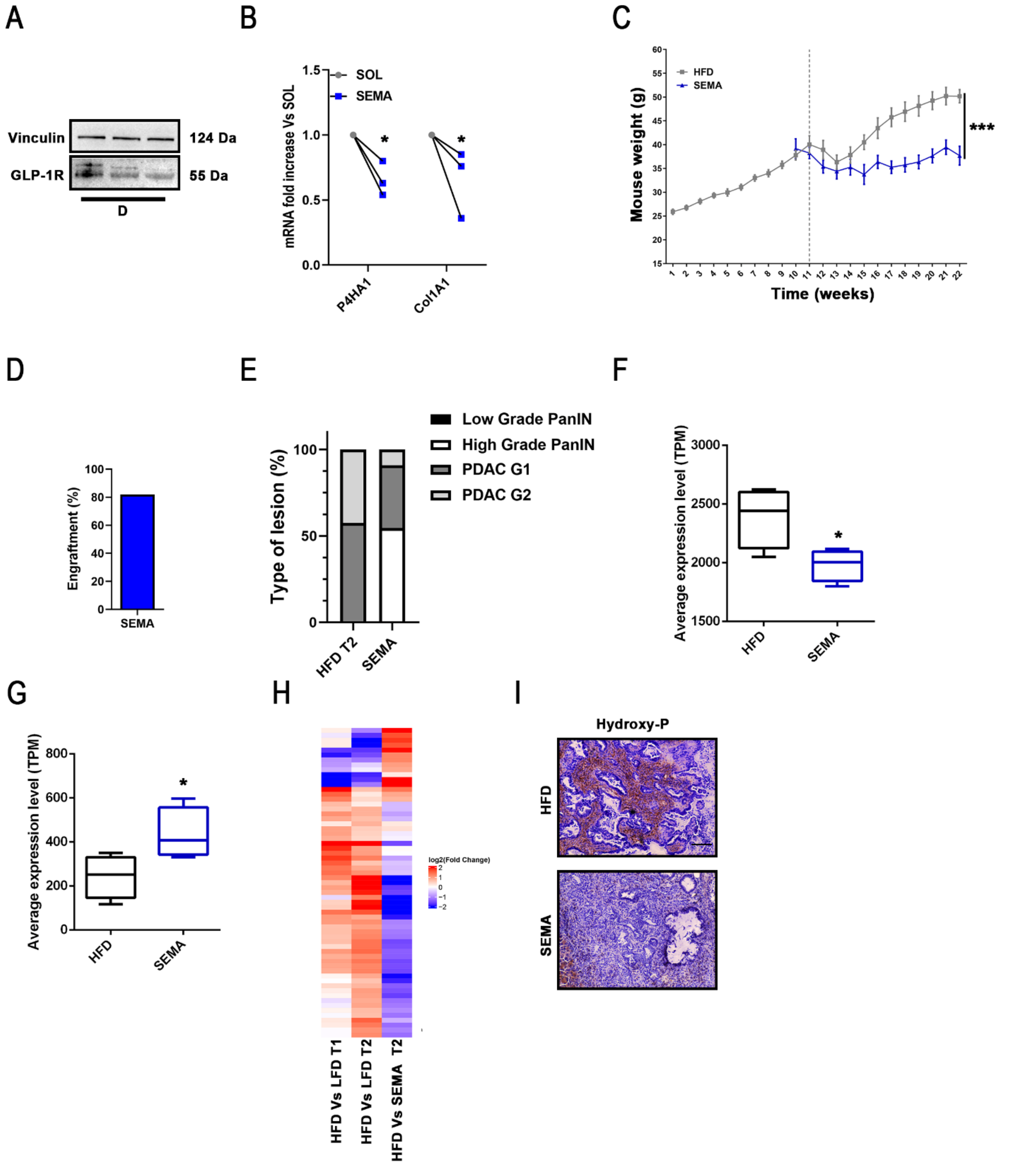

Suppl Fig 5. Semaglutide treatment analysis in D-hpCAFs and HFD mice bearing KPC organoids. A. Representative WB analysis of GLP-1R in D-hpCAFs. Loading control: vinculin (n = 5 for each group). **B.** P4HA1 and Col1A1mRNA analysis in hpCAFs isolated from diabetic PDAC patients upon solvent control (SOL) or 48h semaglutide treatment (SEMA). Data expressed as fold increase of SOL after subtraction of the housekeeping gene p0 signal (n = 3; *$p < 0.05$ SEMA vs. SOL D-hpCAFs). **C.** Animal body weight in HFD (grey squares) and HFD treated with semaglutide (SEMA; blue rumbles) mice bearing pre-neoplastic lesions derived from

pancreatic organoids of KPC mice (n = 18 for each group; ***$p<0.001$). **D.** KPC organoid engraftment evaluation in HFD and SEMA mice after one month from injection into pancreata. **E.** Tumor score of pancreatic cancer progression quantification assigned by a pathologist specialized in pancreatic cancer histology blinded to treatment (HFD Vs SEMA mice; n = 18 for each group). **F.** Boxplots represent the distribution of the average expression level of mesenchymal stromal cell signature in HFD (n = 4; black) and SEMA (n = 3; blue) mice. **G.** Boxplots represent the distribution of the average expression level of T cell signature in HFD (n = 4; black) and SEMA (n = 3; blue) mice. **H.** Heatmap showing a list of selected genes according GO analysis in LFD and HFD mice at time point T1 and T2 and SEMA mice identified by total RNA sequencing analysis (n=3 for LFD T1 and SEMA group; n=4 for LFD T2; HFD T1 and T2 group). Red and blue represent over- and under-expressed genes, respectively. **I.** Representative immunohistochemistry images depicting HFD (upper panels) and SEMA (lower panels) mouse cryosections at time point T2 probed by an anti-hydroxyproline antibody. Nuclei were counterstained with hematoxylin. Merged images depicted. Original scale bar, 100 μm; n = 5. Data expressed as average±SEM. Data analyzed by Wilcoxon test (B) or 2-way-ANOVA (C) or Tukey's HSD after one-way-ANOVA (F and G).

Supplementary Table 1. Characteristics of patients enrolled in the study with relevant clinical features.

Patient (n)	Age (mean+SD)	Gender	Dysmetabolism	BMI (mean+SD)	Therapies
14	62.8±14.7	Male (n=5) Female (n=9)	No	ND+NOb (n=14) 22.4±1.8	
16	62.5±8.0	Male (n=11) Female (n=5)	Yes	ND+Ob (n=2) 35.5±0.4 D+NOb (n=5) 22.0±1.7 D+Ob (n=9) 33.0±3.5	Metformin, Insulin, Dapagliflozin

Nd: no Type II diabetes; D: Type II diabetes; NOb: no obesity; Ob: obesity

Supplementary Table 2. Antibody list.

Antibody name	Company	Cat. Number	Host	Clonality	Application
pAkt	Cell signaling	9271S	Rb	pAb	WB
Akt	Cell signaling	4691S	Rb	mAb	WB
Vinculin	Millipore Sigma	V9131	Ms	mAb	WB
alpha-tubulin	Cell signaling	3873S	Ms	mAb	WB
CD3	Abcam	ab16669	Rb	mAb	IF
PanCK	Invitrogen	MA5-13203	Ms	mAb	IF
E-cad	eBioscience	14-3249-82	Rat	mAb	IF
P4HA1	Abcam	ab244400	Rb	pAb	WB, IF
aSMA	eBioscience	14-9760-82	Ms	mAb	IF
Hydroxyproline	Cell signaling	73812	Rb	pAb	IF,IHC
Col1A1	Santa Cruz	sc-293182	Ms	mAb	IHC
CD8	Abcam	ab217344	Rb	mAb	IF
Myc-Tag	Cell signaling	2276	Ms	mAb	WB
GLP-1R	Bioss	bs-1559R	Rb	pAb	WB
CD29-PB	BD Biosciences	743783	Ms	mAb	FC
CD44-APC	Abcam	ab81424	Ms	mAb	FC
EpCAM-PC	Abcam	ab157319	Ms	mAb	FC
CD31-PB	BioLegend	303113	Ms	mAb	FC
CD45-PB	eBioscience	48-0451-82	Rat	mAb	FC

Ms=mouse; Rb=rabbit; mAb=monoclonal antibody; pAb=polyclonal antibody; WB=western blotting; IF= immunofluorescence; IHC=immunohistochemistry; FC= flow cytometry.

Supplementary Table 3. Primer list.

GENE NAME	FW SEQUENCE	RV SEQUENCE	SP
**Col1A1**	GAGAGAGCATGACCGATGGATT	GGGCCAATGTCTAGTCCGAAT	Mm
**Col4A1**	AGACCATTCAGATTCCGCAGTG	ATGGGGCGCTTCTAAACTCT	Mm
**Col6A1**	GGCCTTGAGGCTGAAACCTTAT	CGAAGTACTTGACCGCATCCA	Mm
**Col1A1**	TGTTCAGCTTTGTGGACCTCCG	ATCCTTAAAAGCTCGCCTGC	Hs
**Col3A1**	TTGGGATTGCTGGGATCACT	GTACCAGCCAGACCAGGAAG	Hs
**Col5A1**	AGCGACCACCCCATGATC	TGGATCCTACAGAAACGACCA	Hs
**P4HA1**	GCCGAGCTACAGTACATGACC	ACCTGTAATTCCTCTGCTGTGG	Hs
**P0**	GCGTCCTGGCATTGTCTGT	GAAGGCCTTGACCTTTTCAGTAAG	Mm
**P0**	TCGACAATGGCAGCATCTAC	ATCCGTCTCCACAGACAAGG	Hs

SP= Species; Mm= Mus Musculus; Hs= Homo Sapiens

Supplementary table 4. sgRNA list for CRISPR/Cas9 experiments.

Name	Sequence	PAM
**P4HA1_1**	GTACGAAATGCTGTGCCGTG	ggg
**P4HA1_2**	CCTGAACATCAGAGAGCTAA	tgg
**NTC**	TGCCGAATCAGTCCACGTAC	

NTC= Non targeting control

Supplementary Table 5. Top50 regulated genes (fdr < 0.05) for the contrast of groups (LFD and HFD at T1) depicted in Figure 1B.

ENSEMBL	chr	start	end	strand	Symbol
ENSMUSG00000002289	17	33992724	34000804	-	Angptl4
ENSMUSG00000015468	17	34783242	34807477	+	Notch4
ENSMUSG00000009418	1	135362318	135615843	-	Nav1
ENSMUSG00000046449	X	103121040	103244791	-	Nexmif
ENSMUSG00000033327	17	34879431	34938789	+	Tnxb
ENSMUSG00000004814	5	135598791	135601903	-	Ccl24
ENSMUSG00000056481	19	5118106	5120710	+	Cd248
ENSMUSG00000005611	7	110467473	110581668	-	Irag1
ENSMUSG00000067206	5	73763985	73789869	-	Lrrc66
ENSMUSG00000004098	9	20681346	20726363	-	Col5a3
ENSMUSG00000021023	12	55346362	55429318	+	Prorp
ENSMUSG00000028763	4	137196080	137297941	+	Hspg2
ENSMUSG00000042138	9	37400317	37435921	+	Msantd2
ENSMUSG00000064358	MT	8607	9390	+	COX3
ENSMUSG00000068699	6	29433255	29461882	+	Flnc
ENSMUSG00000020674	12	29987607	30067657	+	Pxdn
ENSMUSG00000031486	8	27575611	27613464	+	Adgra2
ENSMUSG00000038725	15	44320890	44464765	+	Pkhd1l1
ENSMUSG00000097768	16	93589021	93591953	-	2310043M15Rik
ENSMUSG00000024304	18	16721934	16942303	-	Cdh2
ENSMUSG00000028255	3	144709578	144738537	-	Clca1
ENSMUSG00000029675	5	134731447	134776177	-	Eln
ENSMUSG00000041828	11	109916460	109986804	-	Abca8a
ENSMUSG00000064341	MT	2751	3707	+	ND1
ENSMUSG00000109564	9	18406751	18585826	-	Muc16
ENSMUSG00000020642	12	26350963	26465253	-	Rnf144a
ENSMUSG00000004846	5	137015873	137025502	+	Plod3
ENSMUSG00000020241	10	76431596	76459464	-	Col6a2
ENSMUSG00000028883	5	13175381	13652533	+	Sema3a
ENSMUSG00000028032	3	131270529	131349432	+	Papss1
ENSMUSG00000003500	6	29200433	29216363	-	Impdh1
ENSMUSG00000014813	14	69266687	69279253	+	Stc1
ENSMUSG00000051397	6	67511046	67512780	-	Tacstd2
ENSMUSG00000030077	6	103487547	103727172	+	Chl1
ENSMUSG00000030787	7	110449814	110462446	-	Lyve1
ENSMUSG00000032125	9	37313193	37326411	+	Robo4
ENSMUSG00000024593	18	57266162	57430539	+	Megf10
ENSMUSG00000028013	3	133015871	133083996	+	Ppa2
ENSMUSG00000029108	5	57875309	58290572	+	Pcdh7
ENSMUSG00000048572	19	24651372	24659597	+	Tmem252
ENSMUSG00000040260	17	49763050	49871371	-	Daam2
ENSMUSG00000096054	10	4970917	5501482	-	Syne1

ENSMUSG00000024913	19	3634828	3736564	-	Lrp5
ENSMUSG00000026043	1	45350698	45388866	+	Col3a1
ENSMUSG00000028885	4	132460277	132484563	-	Smpdl3b
ENSMUSG00000008845	6	124281615	124307486	+	Cd163
ENSMUSG00000017969	2	167033725	167082524	-	Ptgis
ENSMUSG00000043079	18	60727045	60793214	-	Synpo
ENSMUSG00000044156	6	3457096	3498298	-	Hepacam2
ENSMUSG00000013643	11	58269869	58281554	+	Lypd8

Supplementary Table 6. Top50 regulated genes (fdr < 0.05) for the contrast of groups (LFD and HFD at T2) depicted in Figure 1D.

ENSEMBL	chr	start	end	strand	Symbol
ENSMUSG00000057335	1	176561219	176641633	-	Cep170
ENSMUSG00000068220	15	78810925	78814665	+	Lgals1
ENSMUSG00000021614	13	89803431	89890628	-	Vcan
ENSMUSG00000028364	4	63878022	63965252	-	Tnc
ENSMUSG00000063779	3	106108806	106126823	-	Chil4
ENSMUSG00000051596	5	38433316	38461552	+	Otop1
ENSMUSG00000006154	7	4463673	4483486	+	Eps8l1
ENSMUSG00000028977	4	148888886	149039346	+	Casz1
ENSMUSG00000054690	3	137046828	137137946	+	Emcn
ENSMUSG00000041143	4	138700199	138786482	+	Tmco4
ENSMUSG00000015354	9	95519654	95580149	+	Pcolce2
ENSMUSG00000020451	11	3294256	3359189	-	Limk2
ENSMUSG00000025347	10	128794143	128796857	-	Mettl7b
ENSMUSG00000034731	14	78796190	78970216	-	Dgkh
ENSMUSG00000052727	13	99557954	99653048	-	Map1b
ENSMUSG00000062078	17	10421530	10538783	-	Qki
ENSMUSG00000074445	3	92192724	92198712	+	Sprr2a3
ENSMUSG00000010751	7	143188543	143203398	-	Tnfrsf22
ENSMUSG00000023942	17	45896126	45910532	-	Slc29a1
ENSMUSG00000038459	7	83758564	83801101	-	Abhd17c
ENSMUSG00000024074	17	78507677	78684021	+	Crim1
ENSMUSG00000032528	9	121471782	121502020	+	Vipr1
ENSMUSG00000064080	6	91189437	91249522	+	Fbln2
ENSMUSG00000068876	3	94667376	94693826	-	Cgn
ENSMUSG00000031438	X	138464065	138573894	+	Rnf128
ENSMUSG00000022755	16	56544972	56616218	-	Adgrg7
ENSMUSG00000074738	4	155778799	155780938	+	Fndc10
ENSMUSG00000022367	15	56529023	56557935	-	Has2
ENSMUSG00000028402	4	81196737	81361052	-	Mpdz
ENSMUSG00000034205	14	69846517	69933283	+	Loxl2
ENSMUSG00000063558	1	58069090	58145572	+	Aox1
ENSMUSG00000070473	5	135015068	135016326	+	Cldn3
ENSMUSG00000007655	6	17306334	17341451	+	Cav1
ENSMUSG00000029032	4	154362943	154386133	-	Arhgef16
ENSMUSG00000031488	8	27628801	27664674	-	Rab11fip1
ENSMUSG00000041570	1	136195861	136273842	-	Camsap2
ENSMUSG00000044906	18	70585283	70605580	-	4930503L19Rik
ENSMUSG00000020689	11	104498826	104561302	+	Itgb3
ENSMUSG00000025504	7	140918793	140942933	+	Eps8l2
ENSMUSG00000060012	14	64884714	65047066	+	Kif13b
ENSMUSG00000019767	10	4432502	4512231	+	Ccdc170
ENSMUSG00000053219	10	22034468	22250038	+	Raet1e

ENSMUSG00000089809	5	99365285	99876924	-	A930011G23Rik
ENSMUSG00000041737	9	31337492	31375758	-	Tmem45b
ENSMUSG00000027624	2	156262829	156385134	+	Epb41l1
ENSMUSG00000068893	3	92123227	92164031	+	Sprr2a2
ENSMUSG00000021594	13	69721568	69759561	-	Srd5a1
ENSMUSG00000024803	19	36089361	36097499	-	Ankrd1
ENSMUSG00000030739	7	44255227	44320267	-	Myh14
ENSMUSG00000040212	7	45567447	45570828	-	Emp3

Supplementary Table 7. Top50 regulated genes (fdr < 0.05) for the contrast of groups (HFD and SEMA at T2) depicted in Figure 4E.

ENSEMBL	chr	start	end	strand	Symbol
ENSMUSG00000035202	9	123195992	123291731	+	Lars2
ENSMUSG00000090733	3	90119829	90120958	-	Rps27
ENSMUSG00000119584	17	40157244	40159092	+	Rn18s
ENSMUSG00000039001	2	179899170	179900238	+	Rps21
ENSMUSG00000077714	2	144107899	144108136	-	Snord17
ENSMUSG00000025733	17	26057431	26063825	-	Rhot2
ENSMUSG00000062580	1	135222951	135241516	-	Timm17a
ENSMUSG00000051998	1	133606829	133617846	-	Lax1
ENSMUSG00000092837	14	51044906	51045224	-	Rpph1
ENSMUSG00000016252	2	174302865	174305898	-	Atp5e
ENSMUSG00000024608	18	60880170	60911618	+	Rps14
ENSMUSG00000119776	11	101518192	101518382	+	Gm23849
ENSMUSG00000073702	1	39406923	39410992	+	Rpl31
ENSMUSG00000025362	10	128460403	128462616	-	Rps26
ENSMUSG00000050856	5	108581110	108582314	-	Atp5k
ENSMUSG00000046516	16	38167353	38182659	+	Cox17
ENSMUSG00000119132	5	115628313	115628453	+	Gm24407
ENSMUSG00000087968	1	87704660	87704931	+	Gm25395
ENSMUSG00000088088	4	43492788	43493058	-	Rmrp
ENSMUSG00000046330	1	72750449	72752972	+	Rpl37a
ENSMUSG00000060636	16	32876823	32880559	+	Rpl35a
ENSMUSG00000034892	12	69204496	69205960	-	Rps29
ENSMUSG00000026032	1	58625543	58635123	+	Ndufb3
ENSMUSG00000060981	13	23715220	23715689	+	H4c8
ENSMUSG00000029603	5	120818267	120849992	-	Dtx1
ENSMUSG00000064360	MT	9459	9806	+	ND3
ENSMUSG00000090862	7	115930740	115933430	-	Rps13
ENSMUSG00000039410	4	154400582	154721330	-	Prdm16
ENSMUSG00000059326	19	61212395	61216867	-	Csf2ra
ENSMUSG00000039781	11	119955256	119977653	-	Cep131
ENSMUSG00000096215	16	4825152	4826173	+	Smim22
ENSMUSG00000119895	12	54743567	54743730	+	Gm22513
ENSMUSG00000065353	4	132079633	132079837	-	Snora73b
ENSMUSG00000119520	5	115627518	115627658	+	Gm24265
ENSMUSG00000021076	12	70984631	71011492	+	Actr10
ENSMUSG00000027860	3	102060899	102112009	-	Vangl1
ENSMUSG00000048897	7	79674562	79743921	+	Zfp710
ENSMUSG00000043702	14	26381113	26391038	-	Pde12
ENSMUSG00000033031	16	48814548	48840072	+	Cip2a
ENSMUSG00000119476	11	87313693	87313856	+	Rnu1a1
ENSMUSG00002076161	9	78082585	78082915	-	Rn7sk
ENSMUSG00000073008	X	106299484	106340375	+	Gpr174

ENSMUSG00000028691	4	116542741	116558019	+	Prdx1
ENSMUSG00000041841	15	5146127	5148622	+	Rpl37
ENSMUSG00000063316	11	101333124	101336355	+	Rpl27
ENSMUSG00000097993	1	135036235	135060332	-	Ptprv
ENSMUSG00000007892	9	61820566	61821824	-	Rplp1
ENSMUSG00000054446	6	30639217	30645362	+	Cpa1
ENSMUSG00000026009	1	61017086	61039479	+	Icos
ENSMUSG00000022185	14	54879618	54924388	-	Acin1

Supplementary Table 8. List of regulated genes (fdr < 0.05) belonging to collagen deposition and immune response gene ontology biological processes in HFD Vs LFD at T1 and T2 and sensitive to SEMA treatment depicted in Supplementary Fig. 5H.

Ensembl ID	Chr	Start	End	Strand	Symbol	HFD Vs LFD T1 log2FC	HFD Vs LFD T2 log2FC	SEMA Vs HFD T2 log2FC
ENSMUSG00000020674	12	29987607	30067657	+	Pxdn	1.38	0.90	-0.57
ENSMUSG00000004846	5	137015873	137025502	+	Plod3	0.71	0.19	-0.59
ENSMUSG00000020241	10	76431596	76459464	-	Col6a2	1.89	0.98	0.05
ENSMUSG00000029108	5	57875309	58290572	+	Pcdh7	2.50	2.04	-1.57
ENSMUSG00000026043	1	45350698	45388866	+	Col3a1	1.84	1.30	-0.83
ENSMUSG00000026837	2	27776437	27929526	+	Col5a1	1.60	0.61	-0.51
ENSMUSG00000040136	7	45753947	45829457	-	Abcc8	-3.93	-1.74	2.69
ENSMUSG00000024620	18	61178222	61218133	+	Pdgfrb	0.98	0.49	-0.18
ENSMUSG00000001119	10	76544626	76562002	-	Col6a1	1.59	1.00	-0.03
ENSMUSG00000031957	8	112413151	112417642	-	Ctrb1	-3.74	-1.17	4.69
ENSMUSG00000032374	9	92424276	92490481	+	Plod2	0.83	1.17	-1.41
ENSMUSG00000000555	15	103252713	103275190	-	Itga5	1.13	0.81	-0.82
ENSMUSG00000064080	6	91189437	91249522	+	Fbln2	1.40	1.65	-2.00
ENSMUSG00000031502	8	11248423	11362826	-	Col4a1	0.90	0.67	-0.61
ENSMUSG00000001506	11	94827050	94843868	+	Col1a1	1.54	0.24	0.90
ENSMUSG00000031503	8	11362805	11499287	+	Col4a2	0.86	0.43	-0.11
ENSMUSG00000029287	5	107254436	107437495	-	Tgfbr3	2.52	0.63	1.39
ENSMUSG00000048126	1	90693645	90771693	-	Col6a3	1.44	1.14	-0.53
ENSMUSG00000049422	10	75768964	75773581	+	Chchd10	-1.72	-1.74	1.98
ENSMUSG00000019916	10	59159118	59209126	+	P4ha1	1.05	0.86	-1.93
ENSMUSG00000022450	15	82234341	82238523	-	Ndufa6	-0.90	-0.39	1.00
ENSMUSG00000029661	6	4504814	4541544	+	Col1a2	1.30	0.40	0.77
ENSMUSG00000028641	4	119090112	119106172	+	P3h1	0.97	0.52	0.34
ENSMUSG00000029648	5	147498414	147662821	-	Flt1	1.05	1.48	-1.39
ENSMUSG00000025515	7	141276583	141308430	+	Muc2	-1.94	-1.57	1.24
ENSMUSG00000022824	16	33614407	33640304	+	Muc13	-3.97	-1.84	0.23
ENSMUSG00000033938	8	84293300	84298255	+	Ndufb7	-0.72	-0.16	0.90
ENSMUSG00000030247	6	142510563	142517340	-	Kcnj8	0.99	1.36	-1.58
ENSMUSG00000006931	11	100299282	100305662	-	P3h4	1.03	1.15	-1.08
ENSMUSG00000031871	8	104828257	104871143	+	Cdh5	0.66	0.97	-1.03
ENSMUSG00000034205	14	69846517	69933283	+	Loxl2	0.92	1.22	-1.54
ENSMUSG00000013584	9	71123071	71203525	+	Aldh1a2	0.85	1.27	-1.59
ENSMUSG00000030249	6	142533588	142648041	-	Abcc9	0.67	1.18	-1.15
ENSMUSG00000023243	14	20190125	20231877	-	Kcnk5	-0.96	-1.19	1.24
ENSMUSG00000028364	4	63878022	63965252	-	Tnc	0.98	2.02	-1.69
ENSMUSG00000056758	10	120197180	120312374	-	Hmga2	1.23	3.58	-3.53
ENSMUSG00000048376	13	95738311	95754995	-	F2r	0.85	1.94	-1.61
ENSMUSG00000026042	1	45413481	45542442	-	Col5a2	0.58	0.82	-0.59
ENSMUSG00000046711	17	27775471	27782648	+	Hmga1	0.67	1.39	-1.58

ENSMUSG00000002900	12	31315233	31379643	+	Lamb1	0.63	1.23	-1.21
ENSMUSG00000040152	2	117942357	117957614	+	Thbs1	0.41	0.76	-1.41
ENSMUSG00000022367	15	56529023	56557935	-	Has2	0.48	1.97	-2.73
ENSMUSG00000029338	5	98030642	98178902	-	Antxr2	0.38	1.39	-1.52
ENSMUSG00000037370	10	24513812	24588057	-	Enpp1	-0.31	0.87	-1.26
ENSMUSG00000046318	18	66178373	66435812	-	Ccbe1	0.75	2.48	-2.05
ENSMUSG00000032035	9	32547517	32669116	+	Ets1	0.24	0.80	0.24
ENSMUSG00000021614	13	89803431	89890628	-	Vcan	0.53	2.16	-2.64
ENSMUSG00000020747	11	115656259	115690192	+	Tmem94	0.19	-1.08	2.74
ENSMUSG00000024659	19	20350792	20368308	-	Anxa1	-0.17	0.93	-1.16
ENSMUSG00000020592	12	8821323	8843715	+	Sdc1	0.19	1.01	-1.51
ENSMUSG00000025809	8	129412135	129459681	+	Itgb1	0.14	0.70	-1.01
ENSMUSG00000020689	11	104498826	104561302	+	Itgb3	0.25	1.56	-0.68
ENSMUSG00000031284	X	142301587	142580792	+	Pak3	-0.33	1.27	-2.54
ENSMUSG00000029552	6	17065148	17105827	+	Tes	0.11	0.96	-1.66
ENSMUSG00000055980	1	82210822	82269137	-	Irs1	0.22	1.34	-1.27
ENSMUSG00000035929	17	35598593	35604266	+	H2-Q4	-0.21	-1.74	1.68
ENSMUSG00000000058	6	17281184	17289114	+	Cav2	0.11	0.89	-1.98
ENSMUSG00000073409	17	35643826	35649031	+	H2-Q6	0.19	-2.03	1.84
ENSMUSG00000060550	17	35658131	35662749	+	H2-Q7	0.20	-1.99	1.64
ENSMUSG00000028614	4	107224981	107273543	+	Ndc1	-0.07	0.66	-1.21
ENSMUSG00000023905	17	23894419	23896442	-	Tnfrsf12a	-0.04	1.11	-1.09
ENSMUSG00000016382	X	74829260	74918788	-	Pls3	-0.04	1.17	-1.25
ENSMUSG00000033031	16	48814548	48840072	+	Cip2a	-0.01	0.62	-1.87
